# Creatine/Creatinine Ratio and Myostatin as Biomarkers to Monitor Muscle Function in Duchenne Muscular Dystrophy Patients

**DOI:** 10.1002/jcsm.70320

**Published:** 2026-06-15

**Authors:** Chiara Degan, Roula Tsonaka, Sharon I. de Vries, Nadine A. Ikelaar, Menno van der Holst, Hermien E. Kan, Erik H. Niks, Pietro Spitali

**Affiliations:** ^1^ Department of Biomedical Data Sciences Leiden University Medical Center the Netherlands; ^2^ Department of Human Genetics Leiden University Medical Center the Netherlands; ^3^ Department of Neurology Leiden University Medical Center the Netherlands; ^4^ Duchenne Center the Netherlands; ^5^ Department of Orthopedics, Rehabilitation and Physiotherapy Leiden University Medical Center the Netherlands; ^6^ Department of Radiology Leiden University Medical Center the Netherlands

**Keywords:** biomarkers, creatine, creatinine, duchenne muscular dystrophy, myostatin

## Abstract

**Background:**

Duchenne Muscular Dystrophy (DMD) is characterized by progressive muscle wasting leading to early loss of motor function. Functional tests monitor disease progression and serve as clinical trial endpoints but are influenced by maturation in younger patients, high intra‐patient variability and patient motivation. Clinical milestones, instead, have been used to identify modifiers of disease progression but are rarely used as primary endpoints since clinical trials are usually too short to capture them. Blood biomarkers, that can reflect disease progression and objectively evaluate treatment responses, offer a valuable alternative.

In this study, we investigated whether longitudinal observations of biomarkers myostatin and creatine/creatinine ratio (Cr/Crn) are associated with functional tests, such as 6‐min walk test, North Star Ambulatory Assessment (NSAA), 10‐m walk‐run test velocity (10MWT), Performance of Upper Limb (PUL2.0) and disease milestones like loss of ambulation, overhead reach and hand‐to‐mouth function.

**Methods:**

We used longitudinal data from an observational study on 74 DMD patients followed between 2009 and 2022 with annual visits to the LUMC outpatient clinic, linked to 408 serum samples. Associations between log2‐biomarkers, functional tests and clinical milestones were assessed using linear mixed models and time‐dependent Cox models. A post‐hoc sample size calculation was performed to evaluate whether the biomarkers' lower intra‐patient variability (NSAA SD: 0.79, 10MWT SD: 0.88, log2‐myostatin and log2‐Cr/Crn SDs: 0.57) may improve future clinical trials design.

**Results:**

Lower Cr/Crn and higher myostatin levels were associated with better functional performance and a less rapid decline in ambulation, given fixed treatment and BMI. Children with one‐unit higher log2‐myostatin levels had, on average, 4.73 points higher NSAA and 3.40 points higher PUL2.0 (*p*‐values < 0.001) and were 42% less likely to lose ambulation over the following year. Conversely, children with one‐unit lower log2‐ratio levels had, on average, 11.40 points higher NSAA and 7.18 points higher PUL2.0 (p‐values < 0.001) and were 3.67 times more likely to remain ambulant. We proved that incorporating log2‐myostatin and log2‐Cr/Crn as endpoints could reduce the required sample size for clinical trials by more than half without compromising statistical power. For instance, to detect a yearly drop of 3 points in the NSAA with 80% power, recruitment requires almost 80 participants in a 1:1 randomized trial, compared to a little more than 50 patients for the respective value of log2‐myostatin or log2‐Cr/Crn.

**Conclusions:**

These findings support the potential of myostatin and Cr/Crn as monitoring biomarkers to enhance trial design and endpoints in clinical and interventional trials for DMD.

## 1 Introduction

Duchenne Muscular Dystrophy (DMD) is a rare neuromuscular disorder primarily affecting males, with an incidence of less than 1 in 5000 males and fewer than 1 per million females [1, 2]. It is caused by pathological variants in the *DMD* gene, which lead to delayed motor development due to progressive loss of muscle mass and muscle function, early loss of ambulation and reduced life expectancy. As the disease advances, individuals become increasingly dependent on wheelchair use, typically experiencing loss of ambulation between 10 and 13 years of age in the absence of corticosteroid therapy [1, 3]. Eventually, ventilatory support is also required due to progressive respiratory and cardiac complications.

Functional tests, such as the 6‐min walk test (6MWT), North Star Ambulatory Assessment (NSAA), 10‐m walk‐run test velocity (10MWT) and Performance of Upper Limb (PUL2.0), are used to evaluate disease progression in routine clinical visits and serve as clinical trial endpoints [4, 5, 6]. However, these tests are often affected by patient motivation, particularly in young children and the evaluator's ability to effectively engage and encourage the patient, making reliable data collection challenging [7]. Moreover, many are characterized by a high intra‐patient variability (which might be caused by multiple factors, e.g., activity days before the assessment or patient motivation), which limits the possibility to detect relevant changes in the patients' trajectories. Lastly, the acquisition is time‐consuming; it requires trained personnel and frequent visits to clinical sites. Alternatively, disease milestones such as loss of ambulation (LoA), loss of the ability to reach overhead (OHR) and loss of the ability to bring the hand to the mouth (HTM) are used to identify modifiers of disease progression [8, 9]. While informative, such milestones are not used as primary endpoints in clinical trials, as patients are typically enrolled early in life, and the duration of the study is too short to meet such milestones. Therefore, there is a need for alternative methods of assessment, such as blood biomarkers, which are objective since they are less dependent on patient cooperation, are quicker to collect and can be studied regardless of the age of the patient and the duration of the study.

Multiple studies have indicated that circulating proteins [10] (e.g., myostatin), metabolites [11] (e.g., creatine and creatinine), lipids [12], miRNAs [13] and mRNAs [14] can discriminate patients from healthy controls, making them potential diagnostic markers. Additionally, recent reports have shown that serum protein levels are influenced by factors such as age [15, 16] and corticosteroid treatment [17, 18], highlighting the importance of examining biomarkers in relation to longitudinal functional performance and treatments. This study focuses particularly on two biomarkers, myostatin and the creatine‐to‐creatinine ratio (Cr/Crn), for the following reasons. Myostatin serum levels have been shown to be reduced in dystrophinopathies, e.g., DMD and Becker muscular dystrophy (BMD) patients [19, 20], while Cr/Crn was shown to be elevated [11, 21] with respect to healthy controls. Furthermore, both biomarkers have been found to be cross‐sectionally associated with DMD clinical performance [11, 19] and associated with disease progression measured by timed tests and clinical scales in patients with BMD [22]. These associations are likely due to the fact that both myostatin levels and the conversion of creatine into creatinine depend on the amount of muscle tissue [23, 24]. Myostatin is a negative regulator of muscle growth, primarily expressed in muscle tissue [25]. Creatine, synthesized by the liver and used as energy buffer during muscular contractions, is nonenzymatically metabolized in skeletal muscle into creatinine, which is then cleared by the kidneys [26]. While creatine and creatinine alone are confounded by diet and kidney function, the ratio between these metabolites can be used to study the reduced conversion of creatine into creatinine. Such reduction can be visualized as an increase in Cr/Crn as muscle mass is lost.

For all these reasons, we hypothesized that myostatin and Cr/Crn could be associated with longitudinal clinical performance in DMD patients. Furthermore, we aimed to determine whether their limited variability, independent of patient motivation, makes them a valuable endpoint for future therapeutic and clinical trials. To address these hypotheses, we investigated serum concentrations of these biomarkers in a retrospective longitudinal cohort of DMD patients followed up at Leiden University Medical Center (LUMC). We associated these serum samples with functional tests and clinical milestones obtained during outpatient visits.

## 2 Methods

### 2.1 Data Collection

Data were collected from DMD patients monitored at LUMC between 2009 and 2022 through the national disease registry for dystrophinopathies, the Dutch Dystrophinopathy Database [27]. Inclusion criteria were solely based on the confirmed genetic diagnosis of DMD, allowing patients to enter the study at different disease stages. Clinical data and serum samples were collected at the same clinic and on the same visit.

Patients participated in yearly visits at the outpatient clinic, during which motor function tests, i.e., 6MWT, NSAA, 10MWT and PUL2.0, were administered by a trained paediatric physiotherapist. Whenever the test was not performed due to factors such as the patient's unwillingness, young age or planning issues, missing values were registered. For 6MWT, NSAA and 10MWT, the values were set to zero at the first non‐ambulant visit and to missing afterwards.

Clinical milestones such as LoA, OHR and HTM were also recorded during the study. LoA was reported by patients and/or caregivers and defined as the moment the individual was first unable to walk 5 m unaided at home. In the absence of such a report, LoA was determined by the clinician as the inability to perform the 10MWT without support. In contrast, OHR and HTM were primarily assessed using the PUL2.0 and the Brooke and Vignos scores.

Data included the patient's age at the time of the visit, body mass index (BMI) and information regarding the corticosteroid used. The treatment variable indicated whether or not the patient was taking corticosteroids (CS) at each specific visit (yes/no). Patients in the study were treated with different corticosteroids (prednisone, deflazacort or vamorolone). Given the retrospective nature of the study, changes in CS type and regimen (daily or intermittent) were present over time for individual patients.

### 2.2 Blood Sampling

Serum samples were collected and prepared according to standard phlebotomy procedures. Samples at LUMC were left to clot for ∼30 min, followed by 10 min of centrifugation at 2350 g. Sample aliquots were frozen at −20°C for 1–2 months and then transferred to −80°C for long‐term storage.

Creatine and creatinine were quantified by UPLC‐MS/MS [28]. Stable isotope‐labelled internal standards were added to 50 μL of serum. A volume of 500 μL of acetonitrile was added while vortexing, followed by centrifugation for 10 min at 12 000 × g. The supernatant was taken to dryness under a nitrogen flow, and the residue was reconstituted in 100 μL of water with 0.01% heptafluorobutyric acid (HFBA). An aliquot of 10 μL of the final solution was injected into a UPLC‐MS/MS (Acquity Xevo TQ‐XS, Waters, Milford, Massachusetts, USA) operating in positive ESI mode using MRMs for the preselected analytes. Separation was achieved on a BEH‐C18 column (100 × 2.1 mm, 1.7 μm) using a linear gradient between solution B (acetonitrile/water, 4:1, v/v) and solution A (0.1% HFBA). The gradient (0.5 mL/min) was as follows: 0–2 min 95% A, 2–3 min 0% A, 3–3.1 min 95% A and 3.1–5 min 95% A for re‐equilibration. All gradient transitions were linear, and the total analysis time, including equilibration, was 5 min. Data processing was performed using MassLynx software (v4.2, Waters, Milford, Massachusetts, USA).

Myostatin was analysed using a Quantikine ELISA kit (Bio‐Techne #DGDF‐80) in combination with a sample activation kit (BioTechne #DY010) and a quality control set 923 (BioTechne #QC98). In brief, 30 μL of serum was activated and subsequently processed and measured in duplicate according to Bio‐Techne protocols using an ID3 plate reader at 450 nm, with a correcting wavelength at 570 nm [20]. Concentrations in pg/mL were calculated after averaging and interpolating the log‐transformed values with a Four Parameter Logistic (4PL) regression. The coefficient of variation (CV) did not exceed 15.3% and had a mean of 3.7%.

### 2.3 Statistical Analysis

All the analyses were conducted using log2‐transformed biomarkers. The log2 transformation addresses skewness and helps in the generation of distributions that are better suited to the assumptions of the analysis. Two linear mixed‐effects models (LMM) [29] were estimated to assess the association of log2‐myostatin and log2‐Cr/Crn ratio with age (centred around its mean) and CS treatment (treated/untreated), including patient‐specific random effects. To evaluate the correlation between the two log2‐biomarkers while accounting for the longitudinal structure of the data, we used the fitted values from the respective models and computed Spearman correlations [30]. The correlation was estimated at the mean age (11.84 years), where the data were most densely concentrated, and stratified by CS treatment.

To determine the association between patients' function and serum myostatin and Cr/Crn, we employed LMM with NSAA, 10MWT, 6MWT and PUL2.0 as outcome variables and with patient‐specific random intercepts. The number of available points was insufficient to include a random slope. As time‐dependent predictors, we considered age, CS treatment (treated/untreated) and body mass index (BMI), as these may affect both patients' performances [31] and biomarkers' levels [17]. Myostatin and Cr/Crn were used as predictors alone, as well as in combination. Zero values in the functional scores were maintained to capture the patients' trajectory up to the loss of ambulation.

To examine the possible association of biomarkers with clinical milestones LoA, OHR and HTM, we used time‐dependent Cox models [32]. We included CS treatment and BMI. The significance of associations was evaluated for both biomarkers individually and jointly, with a *p*‐value < 0.05.

Before fitting the models, log2‐biomarkers, BMI and age were centred. Centring guarantees that the intercept represents the mean value of the outcome when all the continuous predictors are at their mean values (log2‐myostatin: 9.53, log2‐Cr/Crn: 2.90, BMI: 21:30 kg/m^2^, age: 11.84 years), making the interpretation more reasonable.

Post hoc sample size calculation based on functional test outcomes and log2‐biomarkers was performed. For the calculation, an 80% power was assumed, and the effect size was defined using the Minimal Clinically Important Difference (MCID) quantities as reported in the literature for all outcomes [33, 34, 35]. To ensure that outcomes and biomarkers were comparable, we standardized (subtracting the mean and dividing by the standard deviation) the functional tests, log2‐myostatin and log2‐Cr/Crn. We utilized the variance estimates obtained from the LMMs implemented on the outcomes and log2‐biomarkers, with age and CS treatment as predictors and subject‐specific random intercepts. The observed variance depended only on the within‐patient variation. For more details on the power analysis, consult Section S1.

## 3 Results

### 3.1 Cohort Characteristics

Data were collected from 74 DMD patients aged 4 to 24 years (mean: 11.84, SD: 4.15) followed for up to 11 years (min: 1, max: 11, median: 5). One patient was considered an outlier and was removed from the analysis. This decision was made based on the trajectories of both myostatin and Cr/Crn. Even though the clinical results were in line with the rest of the patients' group, the values of the biomarkers were largely outside the interquartile ranges (IQR). For comparison, the analysis results with the outlier are shown in Section S2. The remaining 73 patients had a total of 403 visits. Myostatin was quantified in 355 samples, and Cr/Crn in 368 samples. In 35 serum samples, both biomarkers could not be detected due to insufficient sample volume for analysis; for an extra 13 samples only Cr/Crn could be measured. The number of patients and serum samples used in each analysis varied due to missing values in the clinical data, as detailed for each analysis in the following sections.

Section S3 includes visual timelines of patients' functional test performances and illustrations showing how the likelihood of losing ambulation changes with age. Baseline characteristics are provided in Table 1. Since patients entered the study at various ages, the baseline was defined as each patient's first visit. No patients entered the study unable to bring their hand to the mouth, whereas 9 patients could not reach overhead, and 14 were no longer ambulant. Most patients were treated at baseline; 21 were not receiving corticosteroids.

**TABLE 1:** Patients' characteristics at baseline.

Variable	DMD patients (*n* = 73)
Age, years, median (min–max)	7 (4.10–20.10)
CS treatment, n:	
Treated/corticosteroids yes	52
Untreated/corticosteroids no	21
BMI, kg/m^2^, median (min**–**max)	16.70 (11.40–32.37)
Myostatin, pg/mL, median (min**–**max)	1004 (248–3278)
Creatine, μmol/L, median (min**–**max)	102.10 (65.90–158.40)
Creatinine, μmol/L, median (min**–**max)	16.30 (6.27–31.85)
Creatine/creatinine ratio, median (min**–**max)	6.04 (2.69–16.14)
10MWT, m/s, median (min**–**max)	1.79 (0–2.76)
6MWT, m, median (min**–**max)	347 (0–525)
NSAA, points, median (min**–**max)	23 (0–34)
PUL2.0, points, median (min**–**max)	40 (30–42)
Loss of ambulation, n	14
Loss of overhead reach, n	9
Loss hand to mouth, n	0

Abbreviations: 6MWT, 6‐min walk test; 10MWT, 10‐m walk‐run test velocity; BMI, body mass index; CS, corticosteroids; NSAA, North Star Ambulatory Assessment; PUL2.0, Performance of Upper Limb.

At the end of the study, only 6 patients never started CS treatment while 67 received treatment at least once during the observed time. Among those, three patients received daily CS treatment, while 64 were on an intermittent regimen. The mean age of starting corticosteroids was 5.6 years. Overall, the most frequent corticosteroid used by the patients was prednisone (182 serum samples obtained in patients treated with the drug), followed by deflazacort (148 serum samples). Models of the functional scores, including only age, CS treatment and BMI, showed that CS treatment was significantly associated with better function (10MWT: *p*‐value = 0.006; 6MWT: *p*‐value = 0.004; NSAA: *p*‐value = 0.011; PUL2.0: *p*‐value < 0.001), as detailed in Section S4.

The median age of LoA was 11.00 years, followed by OHR (13.3 years) and HTM (16.8 years). By the end of the study, 50 patients had lost ambulation, 41 had lost the ability to perform OHR and 21 had lost HTM. Models excluding biomarkers showed that CS treatment significantly reduced the risk of LoA (*p*‐value = 0.002) (Section S4).

### 3.2 Association of Biomarkers With Age and CS Treatment

**FIGURE 1:**
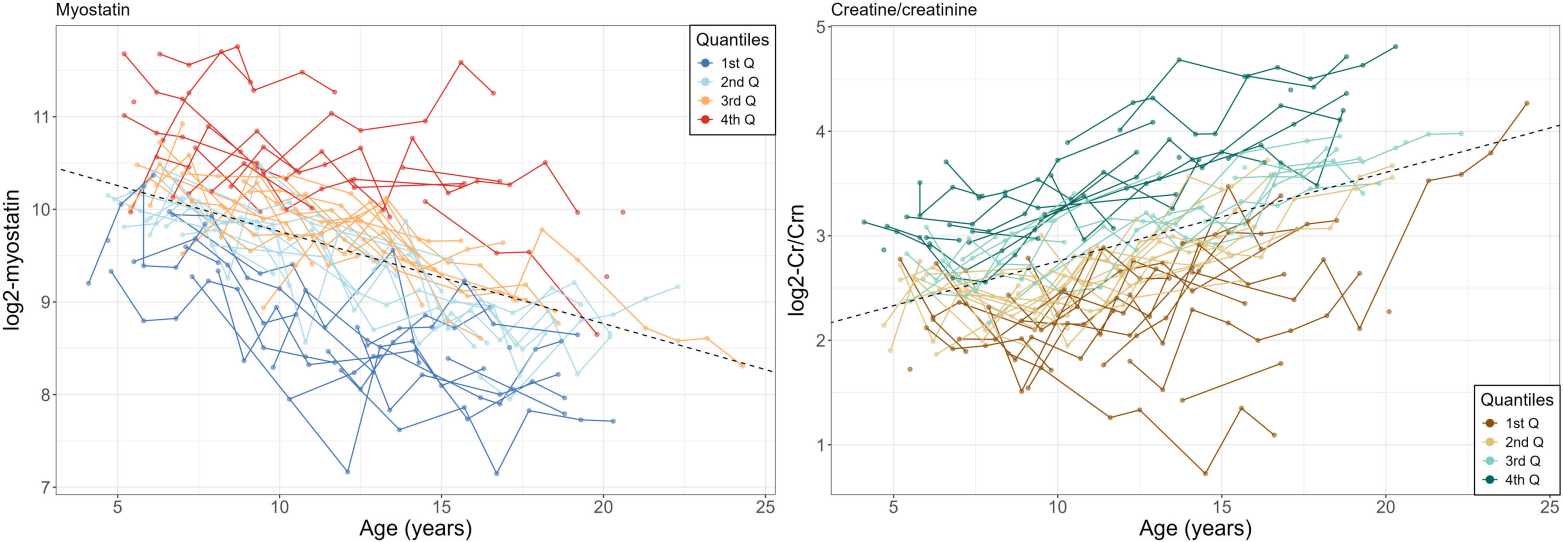
Longitudinal trajectories of log2‐transformed myostatin (left) and creatine/creatinine ratio (right) in DMD patients. Myostatin concentrations were measured in pg/mL and creatine and creatinine in μmol/L. Each line represents a single patient. Colours show whether a patient's overall biomarker levels tend to be higher or lower than the average trend of the study population (dashed line). For myostatin, red lines denote patients whose levels are generally higher than the population average, while blue lines indicate lower‐than‐average trajectories; for the Cr/Crn ratio, teal and brown represent higher and lower trends, respectively. Most patients remain consistently above or below the population average throughout follow‐up, as shown by the clear separation of colours over time. This suggests that, although biomarkers levels decrease (in myostatin) or increase (in the ratio), individual patients tend to preserve their relative position compared with the overall population trajectory. Patient‐specific deviations from the average trend were estimated by using random intercepts from linear mixed‐effects models fitted for each biomarker. Quartiles were used only for illustration and not for statistical modelling. Abbreviations: 1st Q, 1st quartile; Cr/Crn, creatine/creatinine ratio.

The association with age was statistically significant for both myostatin and Cr/Crn (Section S5). Within the same treatment group, the model results estimated that (on average) log2‐myostatin levels decreased by 0.10 units (*p*‐value < 0.001), while log2‐Cr/Crn values increased by 0.08 units (*p*‐value < 0.001) for each year of age (Figure 1).

Across all ages, log2‐myostatin was (on average) 0.46 units higher (*p*‐value < 0.001) in treated versus untreated patients, while log2‐Cr/Crn was 0.34 units lower (*p*‐value < 0.001).

The correlation between myostatin and Cr/Crn was consistent across all ages. At the mean age of 11.84 years, we observed a strong and negative correlation (*R* = −0.746, *p*‐value < 0.001), with no evidence of differences when stratified by treatment group.

### 3.3 Association of Biomarkers With Disease Progression

To illustrate the strength and significance of the associations, we focused on model results from two functional tests: the NSAA for the lower limb function and the PUL2.0 for the upper limb function. Model estimates for the other functional tests are provided in Section S6.

Models for NSAA and either myostatin, Cr/Crn or both biomarkers estimated a yearly decline of 2.24–2.80 points (*p*‐values < 0.001). Patients with a higher BMI tended to perform worse on the NSAA, with an average decrease of ~0.80 points per unit increase in BMI (*p*‐value < 0.001). CS treatment did not show consistent and statistically significant effects across the models. Both biomarkers were significantly associated with performance. Specifically, a one‐unit lower log2‐myostatin was associated with an average 4.73‐point lower NSAA score (1st column, Table 2). In contrast, patients with one‐unit higher log2‐Cr/Crn values had, on average, 11.40 points lower NSAA score (2nd column, Table 2). However, when assessing the joint effect of biomarkers, only Cr/Crn remained statistically significant, and the effect size of myostatin diminished (3rd column, Table 2).

**TABLE 2:** Comparison of regression coefficients of the covariates of three different models for NSAA (left part) and PUL2.0 (right part): one with myostatin alone, one with Cr/Crn ratio alone and one with both. The numbers of samples and patients used for the estimation of each model are reported as well. Statistically significant *p*‐values are highlighted in red.

	NSAA						PUL2.0					
	Only myostatin		Only Cr/Crn ratio		Both biomarkers		Only myostatin		Only Cr/Crn ratio		Both biomarkers	
	Estimates	*p*	Estimates	*p*	Estimates	*p*	Estimates	*p*	Estimates	*p*	Estimates	*p*
*Intercept*	4.85	0.036	5.22	0.007	6.09	0.003	25.5	< 0.001	25.51	< 0.001	26	< 0.001
*Age (years)*	−2.8	< 0.001	−2.28	< 0.001	−2.24	< 0.001	−1.76	< 0.001	−1.32	< 0.001	−1.25	< 0.001
*CS treatment (yes)*	0.95	0.642	0.38	0.832	−0.55	0.769	5.36	< 0.001	4.93	< 0.001	4.25	< 0.001
*BMI* *(kg/m* ^*2*^ *)*	−0.86	< 0.001	−0.8	< 0.001	−0.75	< 0.001	−0.24	0.035	−0.24	0.018	−0.18	0.061
*Log2 – myostatin*	4.73	< 0.001			1.83	0.124	3.4	< 0.001			2.11	0.005
*Log2 ‐ Cr/Crn*			−11.4	< 0.001	−10.02	< 0.001			−7.18	< 0.001	−6.22	< 0.001
*Observations*	186		193		186		183		183		183	
*Patients*	58		62		58		55		55		55	

Abbreviations: BMI, body mass index; Cr/Crn, creatine/creatinine ratio; CS, corticosteroids; NSAA, North Star Ambulatory Assessment; PUL2.0, Performance of Upper Limb.

**FIGURE 2:**
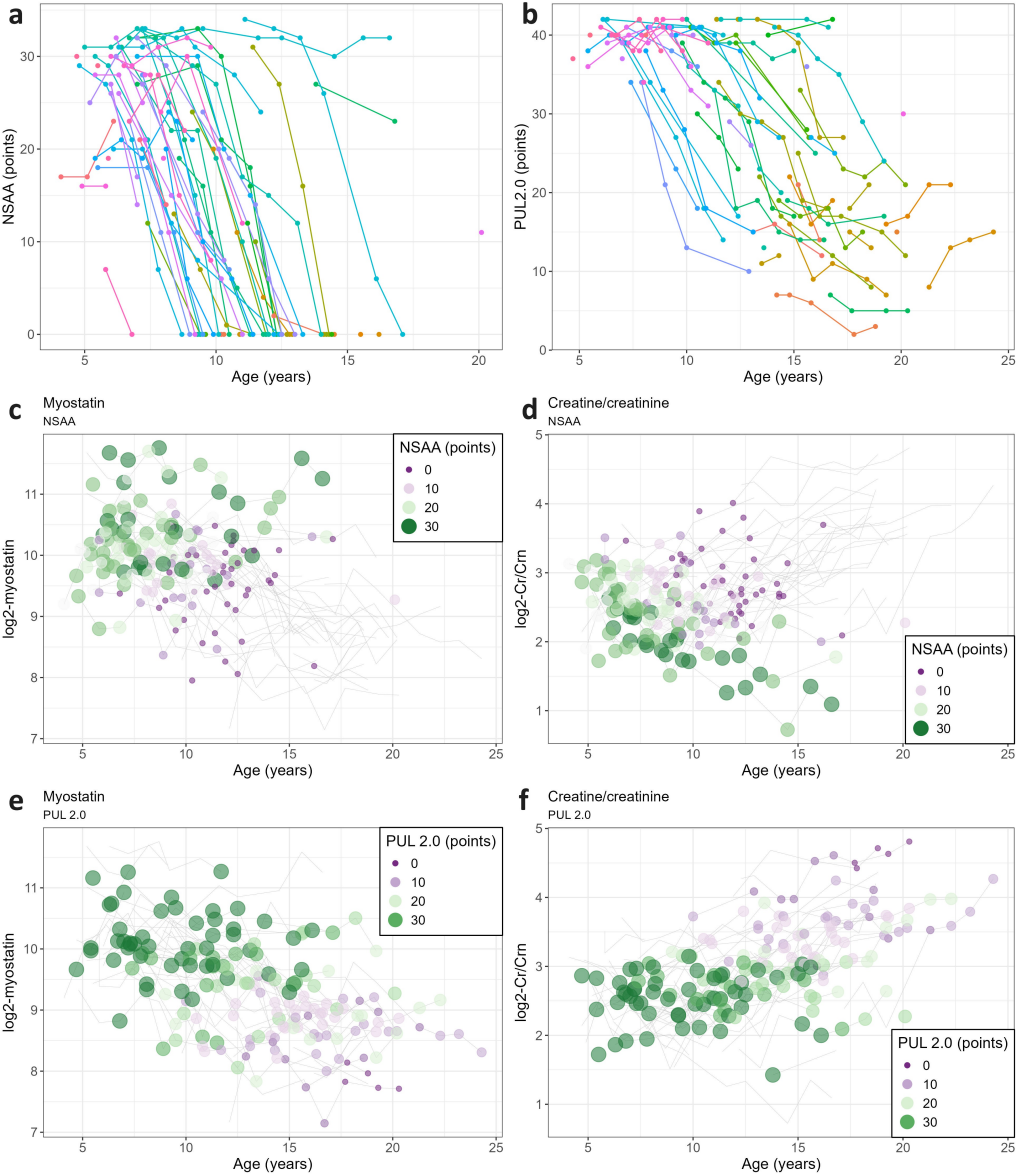
Longitudinal trajectory plots showing the NSAA and the PUL 2.0 scores progressions (panels a and b) with age for all patients. Each line, distinguished by colour, represents an individual patient. In the lower panels c–f, trajectories illustrate how myostatin and Cr/Crn levels (in log2‐scale) relate to NSAA (panels c and d) and PUL 2.0 (panels e and f) scores over time. Each grey line represents an individual patient's biomarker trajectory, and each point corresponds to a clinical visit. Point colour and size encode the functional score at each visit: larger, greener points indicate higher NSAA or PUL 2.0 values, while smaller, more violet points correspond to lower scores. Abbreviations: Cr/Crn, creatine/creatinine ratio; NSAA, North Star Ambulatory Assessment; PUL2.0, Performance of Upper Limb.

The other two lower limb functional scores (6MWT and 10MWT) revealed similar results, supporting this pattern (Section S6). Disease severity increased with age and BMI, meaning that older patients or those with higher BMI on average scored lower on 6MWT and 10MWT. No statistically significant differences in progression were found between treated and untreated patients. There was a statistically significant positive association between myostatin levels and both 6MWT and 10MWT (*p*‐values < 0.001), suggesting that patients with higher myostatin levels tended to perform better on these tests. Conversely, the Cr/Crn ratio showed an opposite pattern: higher Cr/Crn values were associated with poorer performance (*p*‐values < 0.001) (Figure 2).

Similar conclusions were also obtained by modelling PUL2.0 trajectories (Table 2). For PUL2.0, treatment with CS retained a statistically significant effect in all models.

### 3.4 Association of Biomarkers With Clinical Milestones

**FIGURE 3:**
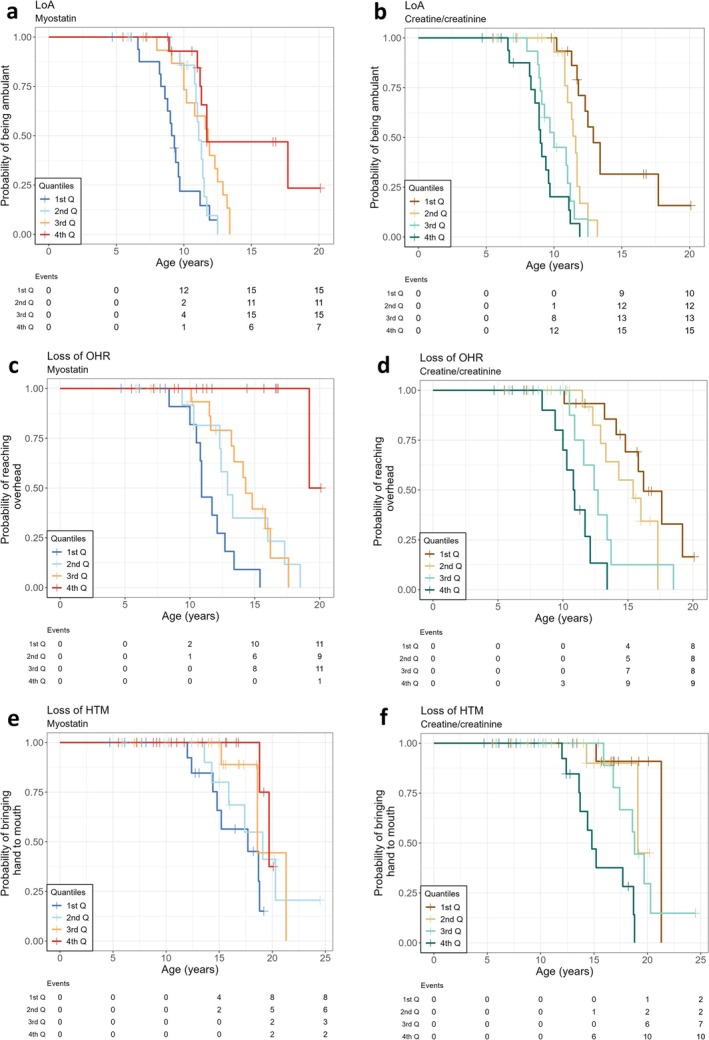
Kaplan–Meier plots showing the relationship between the clinical milestones (LoA: panels a and b, OHR: panels c and d, HTM: panels e and f) and myostatin (left) and Cr/Crn (right). Myostatin and Cr/Crn are log2‐transformed and stratified by quartiles; the higher the quartiles, the higher the patients value of the biomarkers. Quartiles were defined as in Figure 1 and were used only for illustration and not in the statistical modelling. The cumulative number of events (LoA, OHR or HTM) for each age is also reported. Abbreviations: 1st Q, 1st quartile; Cr/Crn, creatine/creatinine ratio; HTM, hand to mouth; LoA, loss of ambulation; OHR, overhead reach.

We sought to assess whether myostatin and Cr/Crn were associated with disease milestones, namely LoA, OHR and HTM (Figure 3). Survival models showed that those with lower log2‐myostatin levels were more likely to lose ambulation within the following year (Table 3). Specifically, patients with a one‐unit lower log2‐myostatin value had a 42% (1–0.58) higher likelihood of losing ambulation, reflecting a more rapid decline in motor function compared to those with higher log2‐myostatin levels (*p*‐value = 0.003). Conversely, patients with one unit lower values of log2‐Cr/Crn were more likely to remain ambulant (Table 3). Subjects having higher log2‐ratio experienced a more rapid decline in ambulation, with their hazard increasing approximately 3.67‐fold (p‐value < 0.001). However, when evaluating the combined effect of both biomarkers, myostatin was no longer statistically significant, while the impact of Cr/Crn on the chance of losing ambulation increased (Table 3). In time‐to‐event models for OHR and HTM, both biomarkers were significantly associated with the outcome but only when myostatin and Cr/Crn were analysed separately (Section S7). BMI was not found to be associated with LoA, while CS treatment significantly decreased the likelihood of LoA only in the model that included myostatin.

**TABLE 3:** Comparison of hazard ratios of the covariates of three different models for LoA: one with myostatin alone, one with Cr/Crn ratio alone and one with both. Statistically significant *p*‐values are highlighted in red.

	Only myostatin		Only Cr/Crn ratio		Both biomarkers	
	HR	*p*	HR	*p*	HR	*p*
*CS* *treatment (yes)*	0.36	0.025	0.56	0.205	0.53	0.173
*BMI (kg/m* ^*2*^ *)*	1.03	0.318	1.06	0.100	1.06	0.086
*Log2‐myostatin*	0.58	0.003			1.40	0.234
*Log2* *‐* *Cr/Crn*			3.67	< 0.001	5.00	< 0.001

Abbreviations: BMI, body mass index; Cr/Crn, creatine/creatinine ratio; CS, corticosteroids; HR, hazard ratios; LoA, loss of ambulation.

### 3.5 Post Hoc Sample Size Calculation

**FIGURE 4:**
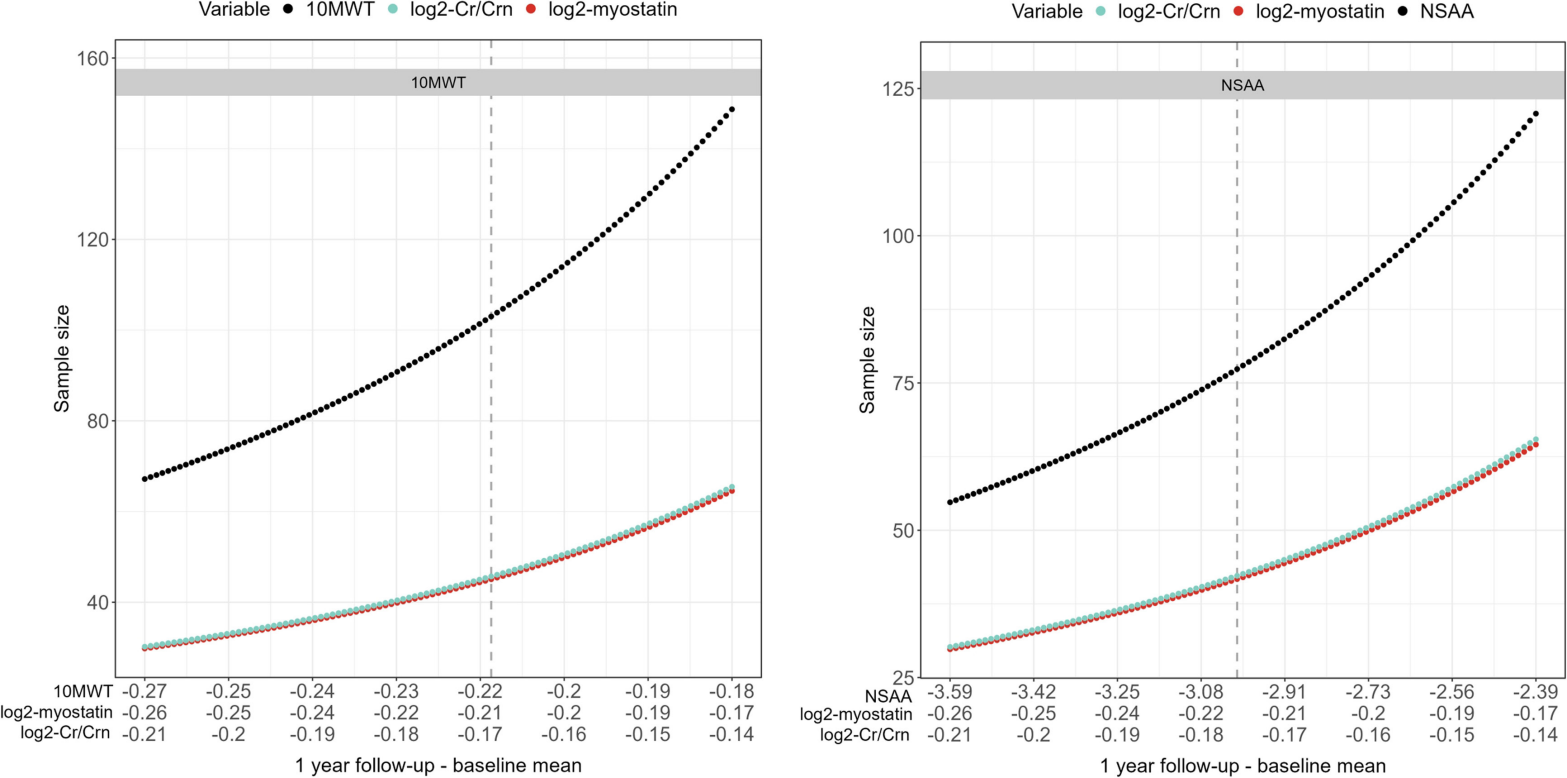
Number of DMD patients needed to detect an effect in 1 year since baseline in a 1:1 randomized trial. Comparison between the number of patients needed to detect the same effect in the 10MWT, in myostatin and in Cr/Crn ratio (left); and the same effect in the NSAA, in myostatin and in Cr/Crn ratio (right). Numbers on the x‐axis are the effect sizes of the corresponding variables. The dashed vertical grey line indicates the point that corresponds, respectively, to the 0.21 m/s decline and the 3‐point drop. Abbreviations: 10MWT, 10‐m walk‐run test velocity; Cr/Crn, creatine/creatinine ratio; NSAA, North Star Ambulatory Assessment.

We wondered whether using biomarkers as endpoints in a hypothetical clinical trial could reduce the required sample size while preserving statistical power. Previous studies in DMD identified a 0.21 m/s decline in 10MWT or a 3‐point decline in NSAA over 1 year as MCID [34, 35]. To detect these effects with 80% power, the required sample size varies depending on the chosen outcome measure and its variability. In this analysis, we estimated the number of subjects needed to detect MCIDs in a clinical trial, accounting for the observed variability of functional tests and biomarkers. Standardized values were used to compare the power analyses based on functional tests or biomarkers. By transforming the standardized values back to the original scales, we were able to determine the changes in log2‐myostatin and log2‐Cr/Crn over 1 year corresponding to a 3‐point decline in NSAA, a 0.21 m/s decline in 10MWT (Figure 4) and a 30‐m decline in the 6MWT (Section S1).

The observed standard deviations between one‐year follow‐up observations of the standardized NSAA and 10MWT were, respectively, 0.79 and 0.88. The within‐patient variances of the standardized biomarkers were instead lower: 0.56 for log2‐myostatin and 0.57 for log2‐Cr/Crn ratio.

The power analysis estimated that almost 80 patients would be needed to observe a 3‐point NSAA decline over 1 year with 80% power in a 1:1 randomized trial, whereas around 50 patients may be sufficient to observe proportional changes in log2‐myostatin or log2‐Cr/Crn levels (Figure 4). A similar trend was observed when comparing the sample sizes needed to detect equivalent effects in biomarkers versus the 10MWT. Using log2‐myostatin and log2‐Cr/Crn as endpoints substantially reduced the number of subjects needed: achieving 80% power to detect an effect equivalent to the MCID of the 10MWT in 1 year would require almost half the number of patients compared to the 10MWT itself.

## 4 Discussion

In this single‐center, retrospective longitudinal study, we used real‐world data to evaluate serum myostatin and the creatine‐to‐creatinine ratio as monitoring biomarkers in DMD. We showed that both biomarkers changed with age, with myostatin progressively decreasing and Cr/Crn correspondingly increasing over time. We showed how these biomarkers were significantly associated with lower and upper limb functional tests and with lower and upper limb disease milestones. Importantly, incorporating these biomarkers as predictors of clinical function attenuated the statistical effect of corticosteroid treatment and eliminated its previously significant association with disease progression. Finally, we showed that a hypothetical trial using Cr/Crn or myostatin as an endpoint would require fewer patients compared to the same trial where functional outcomes are used due to the reduced intra‐patient variation for these biomarkers compared to functional tests.

This study leveraged a large retrospective cohort of 74 DMD patients followed at LUMC for up to 11 years. The long observational time allowed us to confirm how Cr/Crn increases with age, as previously reported by Boca et al. [21] in a cross‐sectional study in 51 patients, and the decrease of myostatin, as reported by Burch et al. [19] in a cross‐sectional comparison of 74 DMD patients. In the same study, Burch et al. [19] collected longitudinal data for a short follow‐up period (3.9 to 18.4 months) in a small sample of 26 DMD patients and could not reveal significant changes in myostatin over that period. Since the number of follow‐up samples was not reported, it is possible that the estimates were affected by the number of longitudinal observations or by the sensitivity of the different assays in Burch et al. [19] work compared to ours.

Our longitudinal study confirmed the previously reported association of these biomarkers with disease severity. Previous cross‐sectional studies showed that Cr/Crn and myostatin were associated with disease severity measured by functional tests such as the 6MWT, NSAA and Forced Vital Capacity (FVC) [11, 19]. We further showed that such associations extend for both biomarkers to the upper limb performance over multiple years. Importantly, in all studies, the directionality of the associations was confirmed, supporting the conclusion that decreasing myostatin and increasing Cr/Crn serum levels are associated with performance decline of lower and upper limb function.

The long follow‐up also enabled the observation of disease milestones such as LoA, OHR and HTM. While previous studies reported differences in these biomarkers between ambulant and non‐ambulant patients [19], we showed that both Cr/Crn and myostatin were significantly associated with the time to event, supporting the idea that they could be used to monitor how changes in these biomarkers are related to the likelihood of experiencing disease milestones.

Interestingly, inclusion of both myostatin and Cr/Crn showed that Cr/Crn ratio was a more stable predictor of function than myostatin. Results of the bivariate model were close to those obtained in the univariate Cr/Crn ratio model, with only small differences due to the effect of myostatin. This can be partly explained by the observed moderate correlation between myostatin and Cr/Crn ratio, justified by the likely relationship of these biomarkers with muscle mass. Such a strong correlation has already been reported in another longitudinal study in BMD patients [22] (*R* = −0.85). Nonetheless, further studies in independent cohorts are needed to clarify the nature of their relationship (confounders, surrogates, etc.) and to validate our findings.

Importantly, the myostatin concentrations measured in this study fall within the previously reported range (0.1–5 ng/mL). In contrast, previous reports of the Cr/Crn only partially align with our results, with all reported values being lower than ours [11, 21, 22] (< 30 for the ratio or < 5 in log2 terms). The observed variability in Cr/Crn distribution may be attributed to differences in the methodology used. While previous studies used relative quantification methods [11, 21], in this study we used an absolute quantification method that provides a quantitative estimation of the levels of creatine and creatinine in serum (μmol/L).

Given the retrospective nature of the data, we corrected for confounding factors such as BMI and CS treatment, which are known to be associated with both function and biomarker and could therefore affect the model estimates of Cr/Crn and myostatin on patients' performance. Our findings support the importance of maintaining a healthy weight for better functional performance. For instance, we observed that across all ages, patients with a BMI that was 1 kg/m^2^ higher tended to have a NSAA score that was approximately 0.80 points lower. However, BMI was not associated with the likelihood of clinical milestones, probably due to the low number of events. Notably, the association between BMI and function remained consistent even after including the biomarkers in the models, suggesting that Cr/Crn and myostatin do not capture differences due to body weight.

Treatment with CS was significantly associated with better function in models without biomarkers, in line with extensive literature supporting the use of corticosteroids to maintain muscle function [31]. Interestingly, when Cr/Crn and myostatin were included in the models, the estimated effect of corticosteroid treatment was reduced and no longer statistically significant. The loss of significance may be due to the progressive reduction in the number of observations over time as patients lose ambulation or to an insufficient number of events within each treatment group in the time‐to‐event analysis. The reduction in CS use estimates could also suggest that myostatin and Cr/Crn could partially capture the beneficial effects of treatment with steroids and function as potential surrogates. However, due to the observational and retrospective nature of the study, the interpretation of these associations should be carefully considered. The effect of CS is confounded by a variety of factors that were not controlled for in our study, such as the age at which treatment was first administered or the type or regimen of corticosteroid taken. Due to these and other factors (such as patients switching between CS medications over time, interruptions of treatment and lean body mass, to mention a few), estimating such a relationship in prospective controlled studies such as FOR‐DMD [31] would provide more reliable estimates. Importantly, our participants followed a 10‐day‐on/10‐day‐off corticosteroids regimen, but no information was available on whether a patient was on the ‘on’ or ‘off’ phase of the CS treatment on the day of the visit/sampling. Additionally, blood sampling and functional assessments were not systematically obtained at the same time of the day, and we did not control for food intake. While more studies are required to confirm whether these biomarkers could eventually function as surrogate endpoints, it is noteworthy that the effect on functional tests of CS treatment may be captured by Cr/Crn and myostatin.

The data showed how Cr/Crn and myostatin could be prospectively used in clinical trials to enrich the design and reduce patient inclusions. While patient inclusions are now based on functional performance, mutation type (depending on the drug) and age ranges, new studies could include patients matching such clinical characteristics and prioritize patients with high Cr/Crn or low myostatin, as these patients represent likely decliners. Our post hoc power analysis showed that using myostatin and Cr/Crn as endpoints can substantially reduce the number of patients needed to power a study. A hypothetical study with 1:1 randomization and 80% power, based on changes in myostatin or Cr/Crn, would halve the number of subjects needed compared to a study powered on clinical outcomes. This finding can be explained by the lower within‐patient variability in biomarker levels compared to functional tests, which provides greater sensitivity in detecting changes across follow‐up observations compared to clinical outcomes. However, these conclusions are based on our observed data and require analysis in independent cohorts to assess whether these biomarkers could be informative during the randomization process or be used as primary, secondary or exploratory endpoints.

In conclusion, we show that Cr/Crn and myostatin provide valuable information as monitoring biomarkers in DMD, as higher Cr/Crn and lower myostatin levels were associated with worse performance and higher risk of disease milestones such as loss of ambulation after adjusting for age, CS treatment and BMI. Moreover, using these biomarkers for patient stratification or as endpoints in future clinical trials may enhance such studies and contribute to reducing the number of subjects needed. Future studies with myostatin and Cr/Crn should aim to narrow the context of use of these biomarkers in DMD and ideally in other neuromuscular conditions, for which initial evidence has been recently reported [36, 37, 38].

## Funding

This study was funded by Duchenne Parent Project NL (Project 22.010). The Dutch Dystrophinopathy Database (DDD) and biobank have been supported by Spieren voor Spieren. This work was supported by the National Institutes of Health under award number # R61NS119639 (Co‐I: Spitali, Tsonaka).

## Consent

Written informed consent was obtained from all participants and/or their legal representatives according to protocol B22.013 at LUMC. The protocol was approved by the regulatory board at LUMC.

## Conflicts of Interest

The authors declare no conflicts of interest.

## Supporting information


**Data S1:** Supplementary Information.

## Data Availability

Anonymized data can be made available to qualified investigators on request. Requests should be in line with the approved ethical protocol.

## References

[1] E. M. Yiu and A. J. Kornberg , “Duchenne Muscular Dystrophy,” Journal of Paediatrics and Child Health 51, no. 8 (2015): 759–764, 10.1111/jpc.12868. 25752877

[2] D. Duan , N. Goemans , S. Takeda , E. Mercuri , and A. Aartsma‐Rus , “Duchenne Muscular Dystrophy,” Nature Reviews. Disease Primers 7, no. 1 (2021): 1–19, 10.1038/s41572-021-00248-3. PMC10557455 33602943

[3] Z. Koeks , C. L. Bladen , D. Salgado , et al., “Clinical Outcomes in Duchenne Muscular Dystrophy: A Study of 5345 Patients From the TREAT‐NMD DMD Global Database,” Journal of Neuromuscular Diseases 4, no. 4 (2017): 293–306, 10.3233/JND-170280. 29125504 PMC5701764

[4] D. N. Shore , “LBA02‐09 EMBARK: A Phase 3 Randomized Study of Enzalutamide or Placebo Plus Leuprolide Acetate and Enzalutamide Monotherapy in High‐Risk Biochemically Recurrent Prostate Cancer,” Journal of Urology 210, no. 1 (2023): 224–226, 10.1097/JU.0000000000003518. 37119051

[5] B. C. Henzi , S. Schmidt , S. Nagy , et al., “Safety and Efficacy of Tamoxifen in Boys With Duchenne Muscular Dystrophy (TAMDMD): A Multicentre, Randomised, Double‐Blind, Placebo‐Controlled, Phase 3 Trial,” Lancet Neurology 22, no. 10 (2023): 890–899, 10.1016/S1474-4422(23)00285-5. 37739572

[6] E. Mercuri , J. J. Vilchez , O. Boespflug‐Tanguy , et al., “Safety and Efficacy of Givinostat in Boys With Duchenne Muscular Dystrophy (EPIDYS): A Multicentre, Randomised, Double‐Blind, Placebo‐Controlled, Phase 3 Trial,” Lancet Neurology 23, no. 4 (2024): 393–403, 10.1016/S1474-4422(24)00036-X. 38508835

[7] L. Alfano , L. Lowes , K. Berry , K. Flanigan , L. Cripe , and J. Mendell , “Role of Motivation on Performance of the 6‐Minute Walk Test in Boys With Duchenne Muscular Dystrophy,” Developmental Medicine and Child Neurology 57, no. S5 (2015): 57–58, 10.1111/dmcn.94_12887.

[8] L. Bello , A. Kesari , H. Gordish‐Dressman , et al., “Genetic Modifiers of Ambulation in the Cooperative International Neuromuscular Research Group Duchenne Natural History Study,” Annals of Neurology 77, no. 4 (2015): 684–696, 10.1002/ana.24370. 25641372 PMC4403971

[9] K. M. Flanigan , M. A. Waldrop , P. T. Martin , et al., “A Genome‐Wide Association Analysis of Loss of Ambulation in Dystrophinopathy Patients Suggests Multiple Candidate Modifiers of Disease Severity,” European Journal of Human Genetics 31, no. 6 (2023): 663–673, 10.1038/s41431-023-01329-5. 36935420 PMC10250491

[10] Y. Hathout , E. Brody , P. R. Clemens , et al., “Large‐Scale Serum Protein Biomarker Discovery in Duchenne Muscular Dystrophy,” Proceedings of the National Academy of Sciences of the United States of America 112, no. 23 (2015): 7153–7158, 10.1073/pnas.1507719112. 26039989 PMC4466703

[11] P. Spitali , K. Hettne , R. Tsonaka , et al., “Cross‐Sectional Serum Metabolomic Study of Multiple Forms of Muscular Dystrophy,” Journal of Cellular and Molecular Medicine 22, no. 4 (2018): 2442–2448, 10.1111/jcmm.13543. 29441734 PMC5867073

[12] N. K. Srivastava , S. Pradhan , B. Mittal , and G. A. N. Gowda , “High Resolution NMR Based Analysis of Serum Lipids in Duchenne Muscular Dystrophy Patients and Its Possible Diagnostic Significance,” NMR in Biomedicine 23, no. 1 (2010): 13–22, 10.1002/nbm.1419. 19787747

[13] M. Llano‐Diez , C. I. Ortez , J. A. Gay , et al., “Digital PCR Quantification of miR‐30c and miR‐181a as Serum Biomarkers for Duchenne Muscular Dystrophy,” Neuromuscular Disorders 27, no. 1 (2017): 15–23, 10.1016/j.nmd.2016.11.003. 27979502

[14] M. Signorelli , M. Ebrahimpoor , O. Veth , et al., “Peripheral Blood Transcriptome Profiling Enables Monitoring Disease Progression in Dystrophic Mice and Patients,” EMBO Molecular Medicine 13, no. 4 (2021): e13328, 10.15252/emmm.202013328. 33751844 PMC8033515

[15] M. Signorelli , B. Ayoglu , C. Johansson , et al., “Longitudinal Serum Biomarker Screening Identifies Malate Dehydrogenase 2 as Candidate Prognostic Biomarker for Duchenne Muscular Dystrophy,” Journal of Cachexia, Sarcopenia and Muscle 11, no. 2 (2020): 505–517, 10.1002/jcsm.12517. 31881125 PMC7113516

[16] K. Strandberg , B. Ayoglu , A. Roos , et al., “Blood‐Derived Biomarkers Correlate With Clinical Progression in Duchenne Muscular Dystrophy,” Journal of Neuromuscular Diseases 7, no. 3 (2020): 231–246, 10.3233/JND-190454. 32390640 PMC7369103

[17] Y. Hathout , C. Liang , M. Ogundele , et al., “Disease‐Specific and Glucocorticoid‐Responsive Serum Biomarkers for Duchenne Muscular Dystrophy,” Scientific Reports 9, no. 1 (2019): 12167, 10.1038/s41598-019-48548-9. 31434957 PMC6704115

[18] U. J. Dang , M. Ziemba , P. R. Clemens , et al., “Serum Biomarkers Associated With Baseline Clinical Severity in Young Steroid‐Naïve Duchenne Muscular Dystrophy Boys,” Human Molecular Genetics 29, no. 15 (2020): 2481–2495, 10.1093/hmg/ddaa132. 32592467 PMC7471506

[19] P. M. Burch , O. Pogoryelova , J. Palandra , et al., “Reduced Serum Myostatin Concentrations Associated With Genetic Muscle Disease Progression,” Journal of Neurology 264, no. 3 (2017): 541–553, 10.1007/s00415-016-8379-6. 28074267

[20] V. Mariot , R. Joubert , C. Hourdé , et al., “Downregulation of Myostatin Pathway in Neuromuscular Diseases May Explain Challenges of Anti‐Myostatin Therapeutic Approaches,” Nature Communications 8, no. 1 (2017): 1859, 10.1038/s41467-017-01486-4. PMC5709430 29192144

[21] S. M. Boca , M. Nishida , M. Harris , et al., “Discovery of Metabolic Biomarkers for Duchenne Muscular Dystrophy Within a Natural History Study,” PLoS ONE 11, no. 4 (2016): e0153461, 10.1371/journal.pone.0153461. 27082433 PMC4833348

[22] N. M. van de Velde , Z. Koeks , M. Signorelli , et al., “Longitudinal Assessment of Creatine Kinase, Creatine/Creatinineratio, and Myostatin as Monitoring Biomarkers in Becker Muscular Dystrophy,” Neurology 100, no. 9 (2023): e975–e984, 10.1212/WNL.0000000000201609. 36849458 PMC9990441

[23] M. Wyss and R. Kaddurah‐Daouk , “Creatine and Creatinine Metabolism,” Physiological Reviews 80, no. 3 (2000): 1107–1213, 10.1152/physrev.2000.80.3.1107. 10893433

[24] A. Mitra , R. Qaisar , B. Bose , and S. P. Sudheer , “The Elusive Role of Myostatin Signaling for Muscle Regeneration and Maintenance of Muscle and Bone Homeostasis,” Osteoporosis and Sarcopenia 9, no. 1 (2023): 1–7, 10.1016/j.afos.2023.03.008. 37082359 PMC10111947

[25] G. Carnac , B. Vernus , and A. Bonnieu , “Myostatin in the Pathophysiology of Skeletal Muscle,” Current Genomics 8, no. 7 (2007): 415–422, 10.2174/138920207783591672. 19412331 PMC2647158

[26] J. T. Brosnan and M. E. Brosnan , “Creatine: Endogenous Metabolite, Dietary, and Therapeutic Supplement,” Annual Review of Nutrition 27 (2007): 241–261, 10.1146/annurev.nutr.27.061406.093621. 17430086

[27] N. M. van de Velde , Y. D. Krom , J. Bongers , et al., “The Dutch Dystrophinopathy Database: A National Registry With Standardized Patient and Clinician Reported Real‐World Data,” Journal of Neuromuscular Diseases 11, no. 5 (2024): 1095–1109, 10.3233/JND-240061. 39031379 PMC11380288

[28] R. S. Carling , S. L. Hogg , T. C. Wood , and J. Calvin , “Simultaneous Determination of Guanidinoacetate, Creatine and Creatinine in Urine and Plasma by Un‐Derivatized Liquid Chromatography‐Tandem Mass Spectrometry,” Annals of Clinical Biochemistry 45, no. Pt 6 (2008): 575–584, 10.1258/acb.2008.008029. 18782816

[29] L. Wu , Mixed Effects Models for Complex Data (Chapman and Hall/CRC, 2009), 10.1201/9781420074086.

[30] M. M. Mukaka , “A Guide to Appropriate Use of Correlation Coefficient in Medical Research,” Malawi Medical Journal: The Journal of Medical Association of Malawi 24, no. 3 (2012): 69. 23638278 PMC3576830

[31] M. Guglieri , K. Bushby , M. P. McDermott , et al., “Effect of Different Corticosteroid Dosing Regimens on Clinical Outcomes in Boys With Duchenne Muscular Dystrophy: A Randomized Clinical Trial,” JAMA 327, no. 15 (2022): 1456–1468, 10.1001/jama.2022.4315. 35381069 PMC8984930

[32] T. M. Therneau and P. M. Grambsch , “Modeling Survival Data: Extending the Cox Model,” in Statistics for Biology and Health (Springer, 2000), 10.1007/978-1-4757-3294-8.

[33] C. M. McDonald , E. K. Henricson , R. T. Abresch , et al., “The 6‐Minute Walk Test and Other Clinical Endpoints in Duchenne Muscular Dystrophy: Reliability, Concurrent Validity, and Minimal Clinically Important Differences From a Multicenter Study,” Muscle & Nerve 48, no. 3 (2013): 357–368, 10.1002/mus.23905. 23674289 PMC3826053

[34] V. A. Gupta , V. Ayyar Gupta , J. M. Pitchforth , et al., “Determining Minimal Clinically Important Differences in the North Star Ambulatory Assessment (NSAA) for Patients With Duchenne Muscular Dystrophy,” PLoS ONE (online) 18, no. 4 (2023): e0283669, 10.1371/journal.pone.0283669. PMC10132589 37099511

[35] T. Duong , J. Canbek , M. Birkmeier , et al., “The Minimal Clinical Important Difference (MCID) in Annual Rate of Change of Timed Function Tests in Boys With DMD,” Journal of Neuromuscular Diseases 8, no. 6 (2021): 939–948, 10.3233/JND-210646. 34151852 PMC8673528

[36] U. Moore , E. Fernández‐Simón , M. Schiava , et al., “Myostatin and Follistatin as Monitoring and Prognostic Biomarkers in Dysferlinopathy,” Neuromuscular Disorders 33, no. 2 (2023): 199–207, 10.1016/j.nmd.2023.01.001. 36689846

[37] A. T. Tebbenkamp , S. B. Huggett , V. Lombardi , et al., “Protein Biomarker Signature in Patients With Spinal and Bulbar Muscular Atrophy,” JCI Insight 9, no. 13 (2024): e176383, 10.1172/jci.insight.176383. 38973610 PMC11383357

[38] A. L. A. de Albuquerque , J. K. Chadanowicz , G. C. Giudicelli , et al., “Serum Myostatin as a Candidate Disease Severity and Progression Biomarker of Spinal Muscular Atrophy,” Brain Communications 6, no. 2 (2024): fcae062, 10.1093/braincomms/fcae062. 38487549 PMC10939446

