# Supplementary material for: Creatine/Creatinine Ratio and Myostatin as Biomarkers to Monitor Muscle Function in Duchenne Muscular Dystrophy Patients
Source: J Cachexia Sarcopenia Muscle. 2026 Jun 15;17(3):e70320. doi: 10.1002/jcsm.70320 (PMC13269173; doi:10.1002/jcsm.70320)
Supplement: Supplementary file 1 — Data S1: Supplementary Information. [file JCSM-17-e70320-s001.pdf]

Creatine/creatinine ratio and myostatin as biomarkers to monitor muscle function in Duchenne Muscular Dystrophy patients – Supplementary material

Chiara Degan, Roula Tsonaka, Sharon I. de Vries, Nadine Ikelaar, Menno van der Holst, Hermien E. Kan, Erik H. Niks, Pietro Spitali

Contents

S1 Post-hoc power analysis 2

S2 Analysis with the outlier 4

S3 Exploration of the data 9

S3.1 Longitudinal progression 11

S3.1.1 Six-minute walk test 12

S3.1.2 North Star Ambulatory Assessment 12

S3.1.3 10-minute walk/run test 13

S3.1.4 PUL 2.0 14

S3.2 Clinical milestones 15

S3.2.1 Loss of ambulation 16

S3.2.2 Loss of overhead reach function 16

S3.2.3 Loss of hand to mouth function 17

S4 Association of functional scores and clinical milestones with CS treatment 19

S5 Association of biomarkers with age and CS treatment 21

S6 Association of the functional tests with the biomarkers 21

S7 Association of the clinical milestones with the biomarkers 22

S8 Association with creatinine 22

References 25

S1 Post-hoc power analysis

The purpose of this post-hoc power analysis is to estimate the number of patients required for a 1:1 randomized clinical trial in which the primary endpoint is alternatively NSAA, 10MWT, 6MWT, myostatin, or the creatine-to-creatinine ratio.

The null hypothesis underlying the power analysis and sample size calculation is:

$$H_0 : \mu_2 - \mu_1 = 0$$

where μ_1 and μ_2 denote, respectively, the mean value of the variable of interest y at age a and $a+1$ (i.e., after one year). The Cohen (Cohen 1988) formula to determine the required sample size for a pairwise t-test is:

$$n = \frac{(Z_{1-\alpha/2} + Z_{1-\beta})^2 \cdot \sigma^2}{\Delta^2},$$

where:

- n is the required sample size;
- $Z_{1-\alpha/2}$ is the critical value for the significance level α (equal to 1.96 for $\alpha = 0.05$);
- $Z_{1-\beta}$ is the critical value for the desired power $1-\beta$ (1.28 for 90% power, and 0.84 for 80% power).
- σ^2 is the variance of the differences between paired measurements, defined as

  $$var(y_2) + var(y_1) - 2cov(y_1, y_2),$$

  where y_1 denotes the vector of observed values of the variable of interest at age a, and y_2 is the vector at age $a+1$.
- Δ is the Minimal Clinically Important Difference (MCID), or the effect the study aims to detect.

This calculation is automatically implemented in the function *pwr.t.test*, in which the sample size n, the desired power, the significance level, and the standardized effect size ($\frac{\Delta}{\sigma}$) must be specified. In this study, the value of $\sqrt{\sigma^2}$ was obtained using the properties of the linear mixed models (LMMs). Let $y_2 = (\hat{y}|age = a+1)$ and $y_1 = (\hat{y}|age = a)$. Under the LMM, the variance of the difference is:

$$var(y_2 - y_1) = var(y_2) + var(y_1) - 2cov(y_1, y_2) = \sigma_\epsilon^2 + \sigma_0^2 + \sigma_\epsilon^2 + \sigma_0^2 - 2\sigma_0^2 = 2\sigma_\epsilon^2.$$

This corresponds to the variance of the marginal predicted values, but the same result is obtained for the conditional predicted values (i.e., conditioning on the random intercept b_0) $y_2 = (\hat{y}|age = a+1, b_0)$ and $y_1 = (\hat{y}|age = a, b_0)$.

The objective is to compare the results obtained from the functional tests (6MWT, NSAA, 10MWT) with those derived from the biomarkers (myostatin and creatine-to-creatinine ratio). The focus lies on assessing how the required sample size varies when the effect size Δ, the statistical power, and the significance level are held constant, but different outcome-specific variances are assumed. The LMMs were estimated on standardized outcomes (subtracting the mean and dividing by the standard deviation), enabling direct comparability across different outcomes.

The results presented below refer specifically to the 6MWT; corresponding analyses for NSAA and 10MWT are provided in the manuscript. As the empirical variances of myostatin (SD: 0.56), creatine-to-creatinine ratio (SD: 0.57), and 6MWT (SD: 0.89) differ substantially. These differences are expected to influence the resulting sample size estimates. To illustrate this effect, the comparison is performed across a sequence of values for Δ. The mean value of this sequence corresponds to the Minimal Clinically Important Difference (MCID) established for the 6MWT, defined as a decline of 30 meters (McDonald et al. 2013).

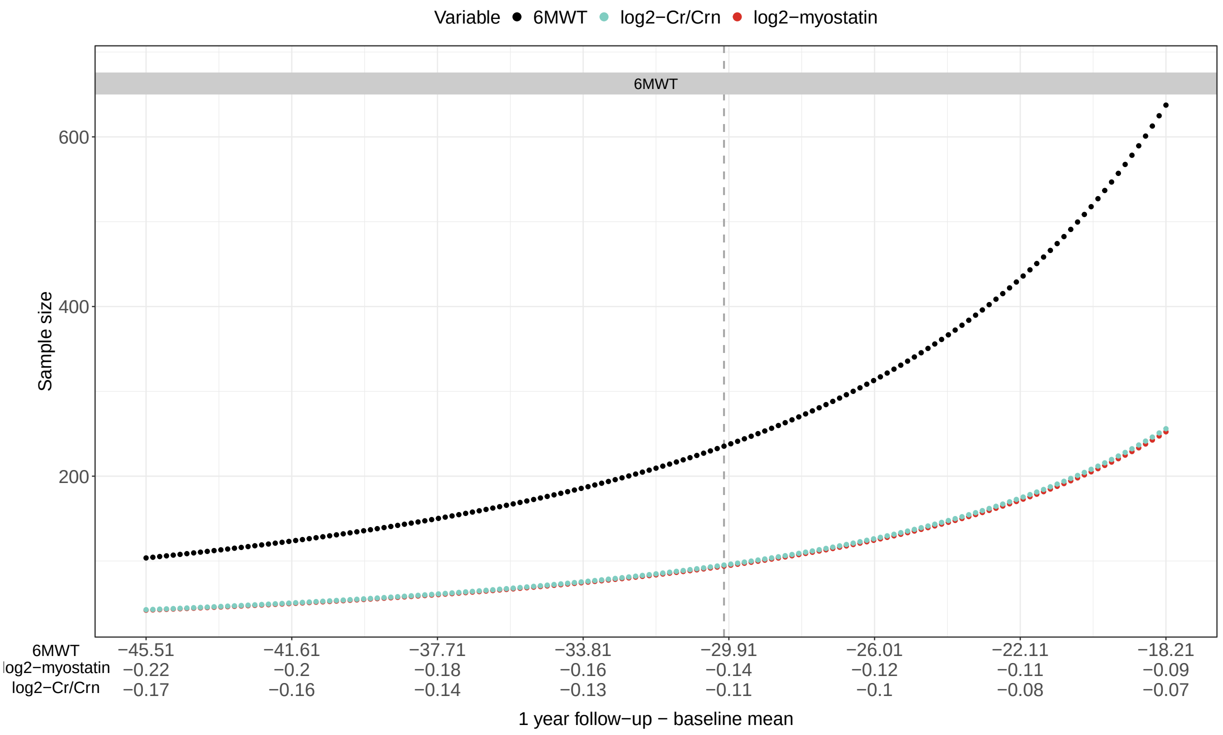
Variable 6MWT log2−Cr/Crn log2−myostatin
6MWT
Sample size
600
400
200
6MWT
log2−myostatin
log2−Cr/Crn
−45.51 −41.61 −37.71 −33.81 −29.91 −26.01 −22.11 −18.21
−0.22 −0.2 −0.18 −0.16 −0.14 −0.12 −0.11 −0.09
−0.17 −0.16 −0.14 −0.13 −0.11 −0.1 −0.08 −0.07
1 year follow−up − baseline mean

S2 Analysis with the outlier

One patient was identified as an outlier and excluded from the analysis. This decision was based on the trajectories of both myostatin and the creatine-to-creatinine ratio (see plots below), which showed atypical patterns compared with the rest of the cohort. In contrast, the trajectories of the functional scores were within the expected range. No information in the clinical records suggested any factor that could have influenced the biomarker measurements. Furthermore, no issues were reported during blood withdrawal or sample processing, and the samples showed no signs of hemolysis.

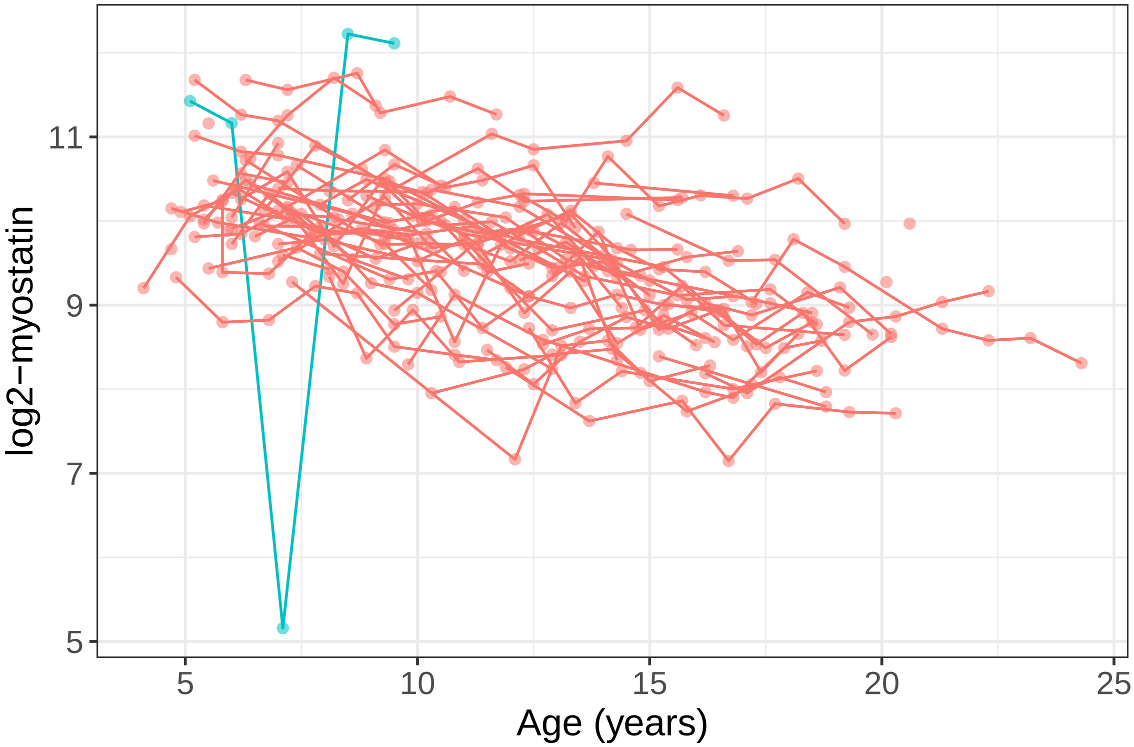

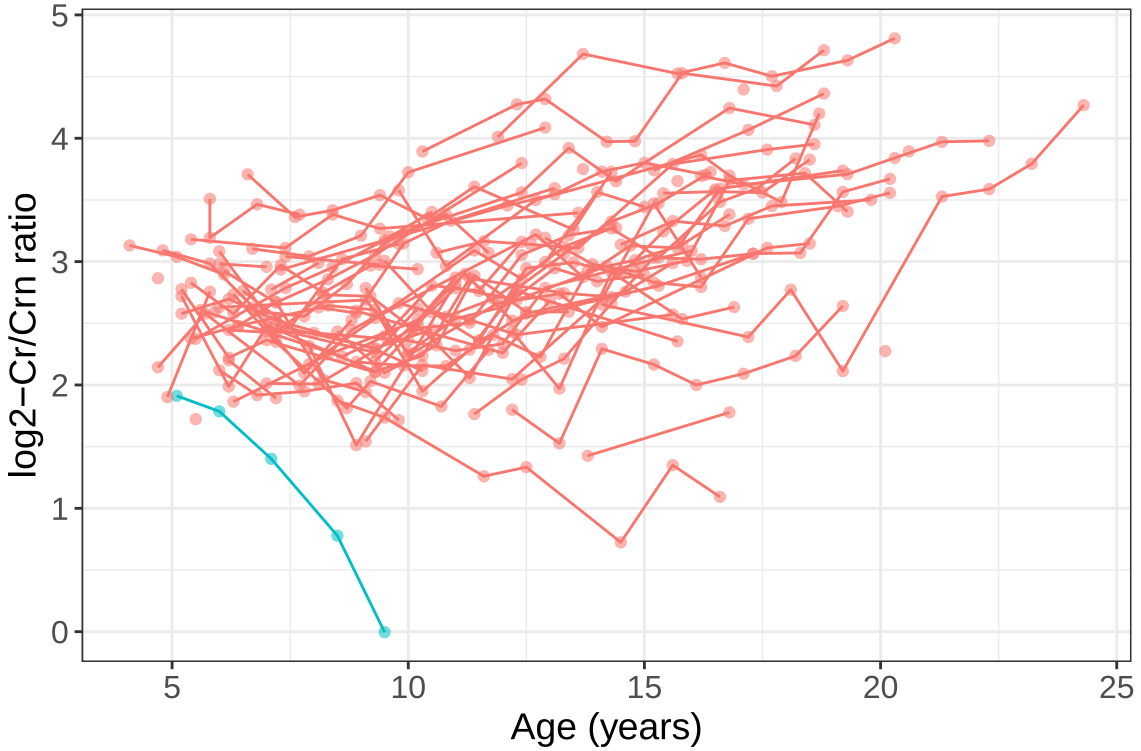

log2−Cr/Crn ratio
Age (years)

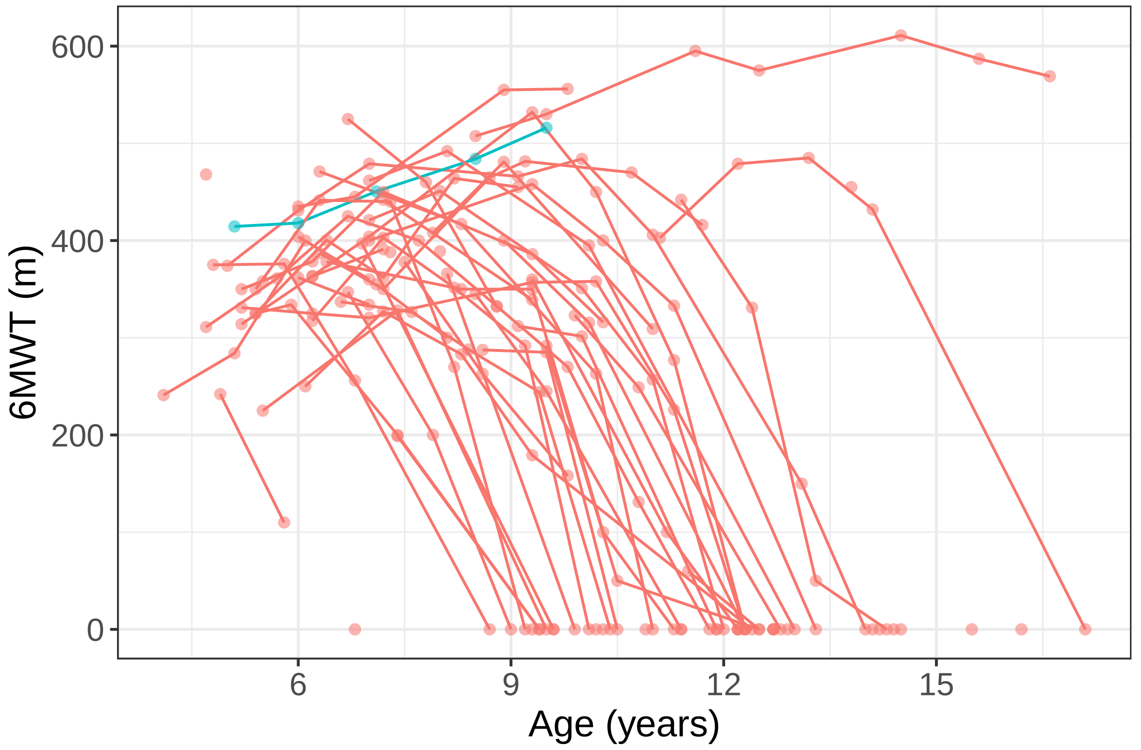

6MWT (m)
Age (years)

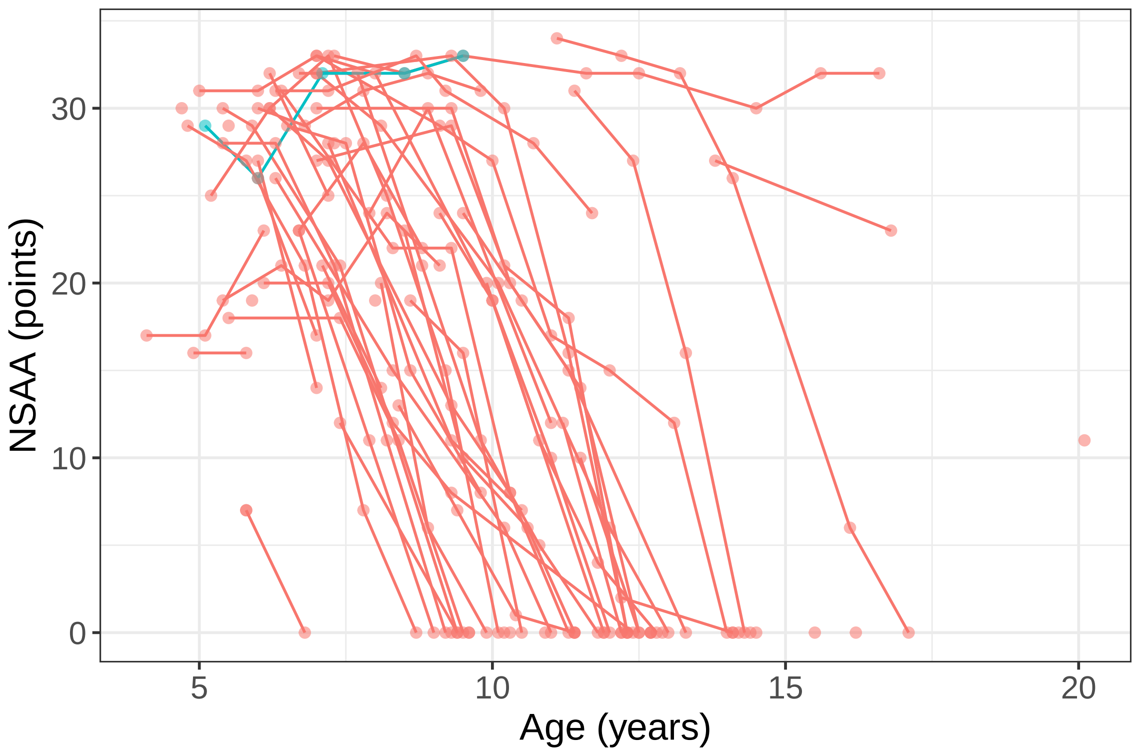
NSAA (points)
Age (years)

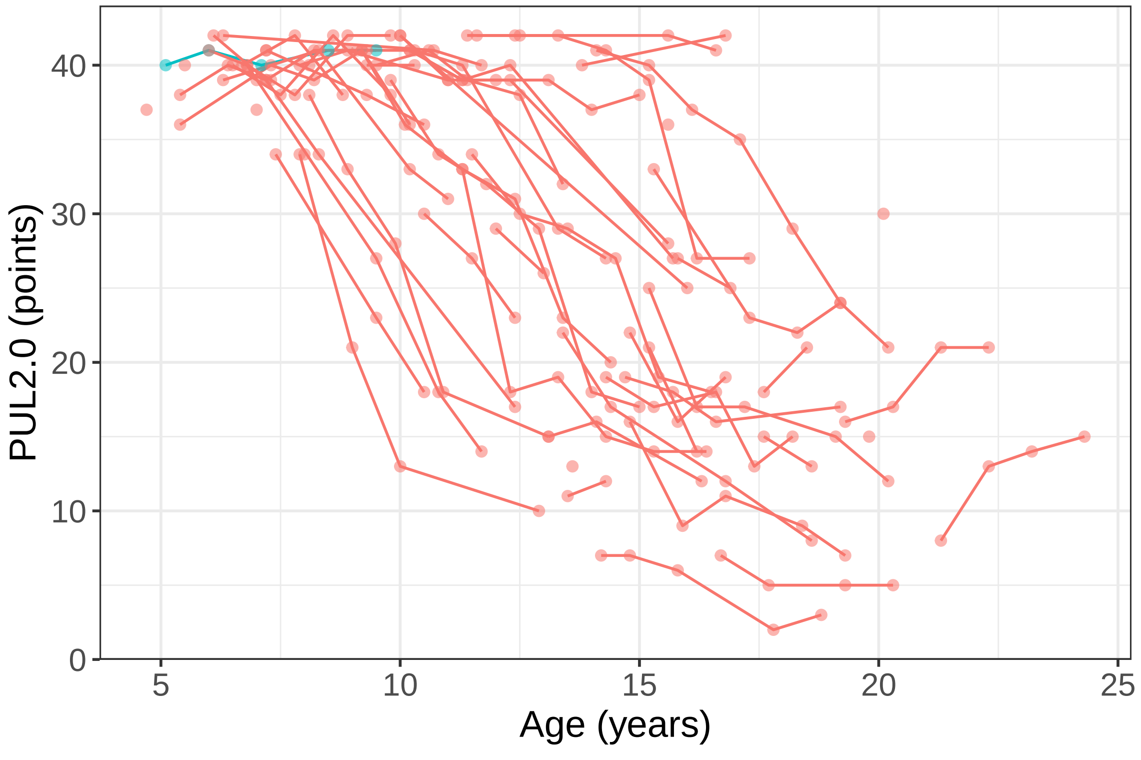
PUL2.0 (points)
Age (years)

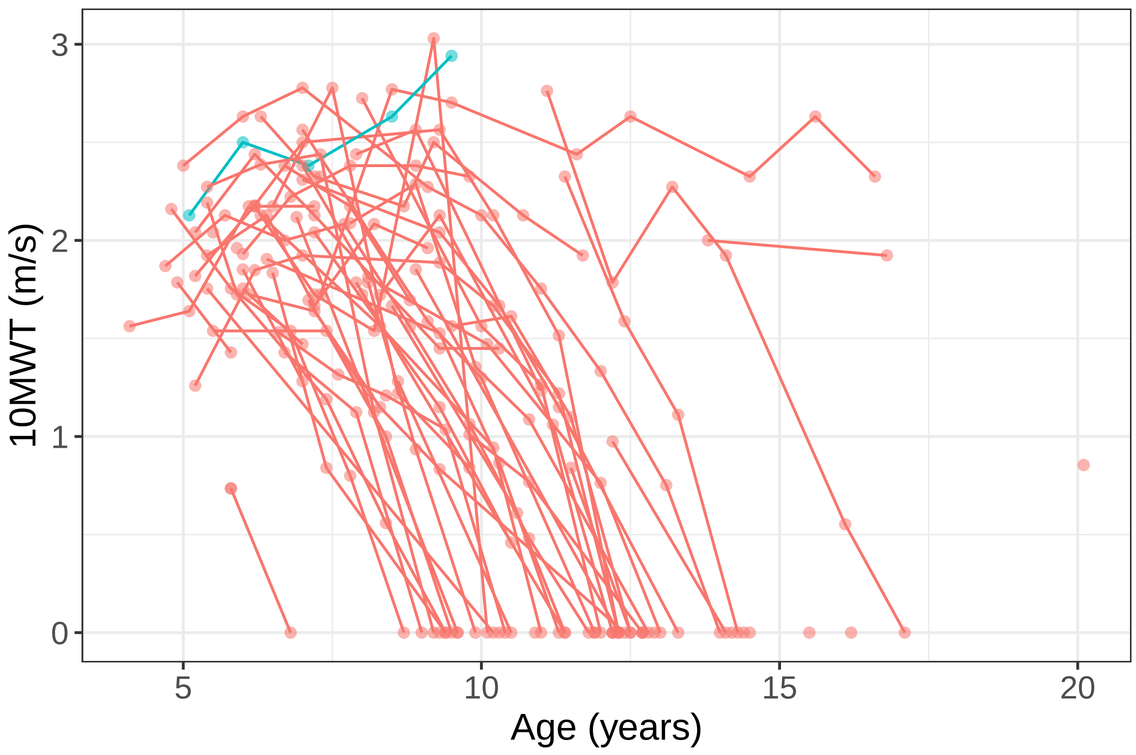

Here we reported the results of the analysis done keeping the patient. The conclusions are the same as in the manuscript, only the magnitude of the estimates in the functional test analysis changed, especially myostatin and age estimates.

Table 1: Comparison of the models between NSAA with only myostatin, with only ratio and with both of them

	Only myostatin		Only ratio		Both	
Predictors	Estimates	p-values	Estimates	p-values	Estimates	p-values
Intercept	3.661	0.114	4.751	0.013	5.237	0.008
Age (years)	-2.865	< 0.001	-2.400	< 0.001	-2.429	< 0.001
Treatment (yes)	3.083	0.114	0.677	0.694	0.284	0.874
BMI (kg/m^2)	-0.969	< 0.001	-0.816	< 0.001	-0.796	< 0.001
log2-myostatin	2.031	0.008			0.487	0.524
log2-Cr/Crn			-10.333	< 0.001	-9.813	< 0.001

Table 2: Comparison of the models between 10MWT with only myostatin, with only ratio and with both of them

	Only myostatin		Only ratio		Both	
Predictors	Estimates	p-values	Estimates	p-values	Estimates	p-values
Intercept	0.458	0.007	0.457	< 0.001	0.494	< 0.001
Age (years)	-0.168	< 0.001	-0.156	< 0.001	-0.149	< 0.001
Treatment (yes)	0.246	0.1	0.091	0.46	0.084	0.513
BMI (kg/m^2)	-0.074	< 0.001	-0.063	< 0.001	-0.059	< 0.001
log2-myostatin	0.193	0.001			0.055	0.336
log2-Cr/Crn			-0.832	< 0.001	-0.793	< 0.001

Table 3: Comparison of the models between 6MWT with only myostatin, with only ratio and with both of them

	Only myostatin		Only ratio		Both	
Predictors	Estimates	p-values	Estimates	p-values	Estimates	p-values
Intercept	104.78	0.004	82.95	0.004	93.456	0.002
Age (years)	-20.74	< 0.001	-22.16	< 0.001	-20.643	< 0.001
Treatment (yes)	61.09	0.06	44.99	0.086	39.225	0.15
BMI (kg/m^2)	-18.08	< 0.001	-15.87	< 0.001	-15.397	< 0.001
log2-myostatin	42.31	0.001			7.866	0.519
log2-Cr/Crn			-169.48	< 0.001	-163.586	< 0.001

Table 4: Comparison of the models between PUL2.0 with only myostatin, with only ratio and with both of them

	Only myostatin		Only ratio		Both	
Predictors	Estimates	p-values	Estimates	p-values	Estimates	p-values
Intercept	24.748	< 0.001	25.615	< 0.001	25.670	< 0.001
Age (years)	-1.877	< 0.001	-1.465	< 0.001	-1.447	< 0.001
Treatment (yes)	6.436	< 0.001	4.821	< 0.001	4.720	< 0.001
BMI (kg/m^2)	-0.322	0.005	-0.260	0.012	-0.239	0.021
log2-myostatin	1.404	0.001			0.786	0.079
log2-Cr/Crn			-5.712	< 0.001	-5.313	< 0.001

Table 5: Comparison of the models between LoA with only myostatin, with only ratio and with both of them

	Only myostatin		Only ratio		Both	
Predictors	Hazard Ratios	p-values	Hazard Ratios	p-values	Hazard Ratios	p-values
Treatment (yes)	0.351	0.020	0.559	0.205	0.535	0.174
BMI (kg/m^2)	1.035	0.294	1.058	0.1	1.059	0.085
log2-myostatin	0.590	0.002			1.405	0.234
log2-Cr/Crn			3.679	< 0.001	5.005	< 0.001

Table 6: Comparison of the models between OHR with only myostatin, with only ratio and with both of them

	Only myostatin		Only ratio		Both	
Predictors	Hazard Ratios	p-values	Hazard Ratios	p-values	Hazard Ratios	p-values
Treatment (yes)	0.805	0.631	1.653	0.309	1.642	0.321
BMI (kg/m^2)	0.986	0.73	1.024	0.566	1.024	0.576
log2-myostatin	0.445	< 0.001			0.969	0.926
log2-Cr/Crn			7.172	< 0.001	6.897	0.002

Table 7: Comparison of the models between HTM with only myostatin, with only ratio and with both of them

	Only myostatin		Only ratio		Both	
Predictors	Hazard Ratios	p-values	Hazard Ratios	p-values	Hazard Ratios	p-values
Treatment (yes)	0.561	0.425	0.659	0.583	0.678	0.614
BMI (kg/m^2)	1.004	0.937	1.019	0.721	1.021	0.700
log2-myostatin	0.367	0.057			0.819	0.796
log2-Cr/Crn			4.188	0.018	3.553	0.149

S3 Exploration of the data

The analysis has been done on data of 73 Duchenne Muscular Dystrophy patients (403 serum samples).

In the following plot, the treatment variable indicates whether or not the patient is taking corticosteroids (CS) at each specific visit (yes/no), as the situation may change over time depending on the effects or side effects of the steroids on the patient. The dashed line indicates the mean age of starting corticosteroids (5.68 years).

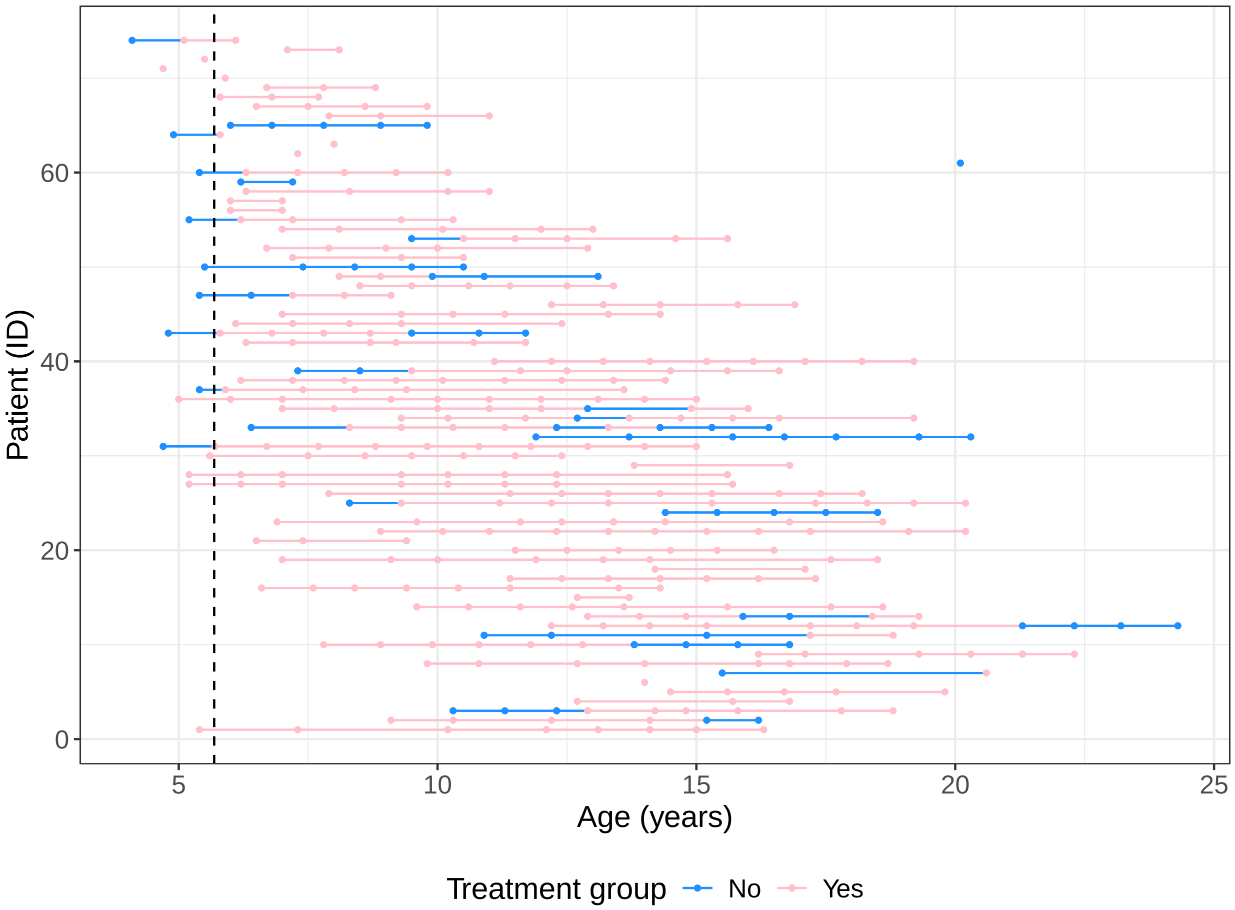

The type of corticosteroids used by the patients changed over time:

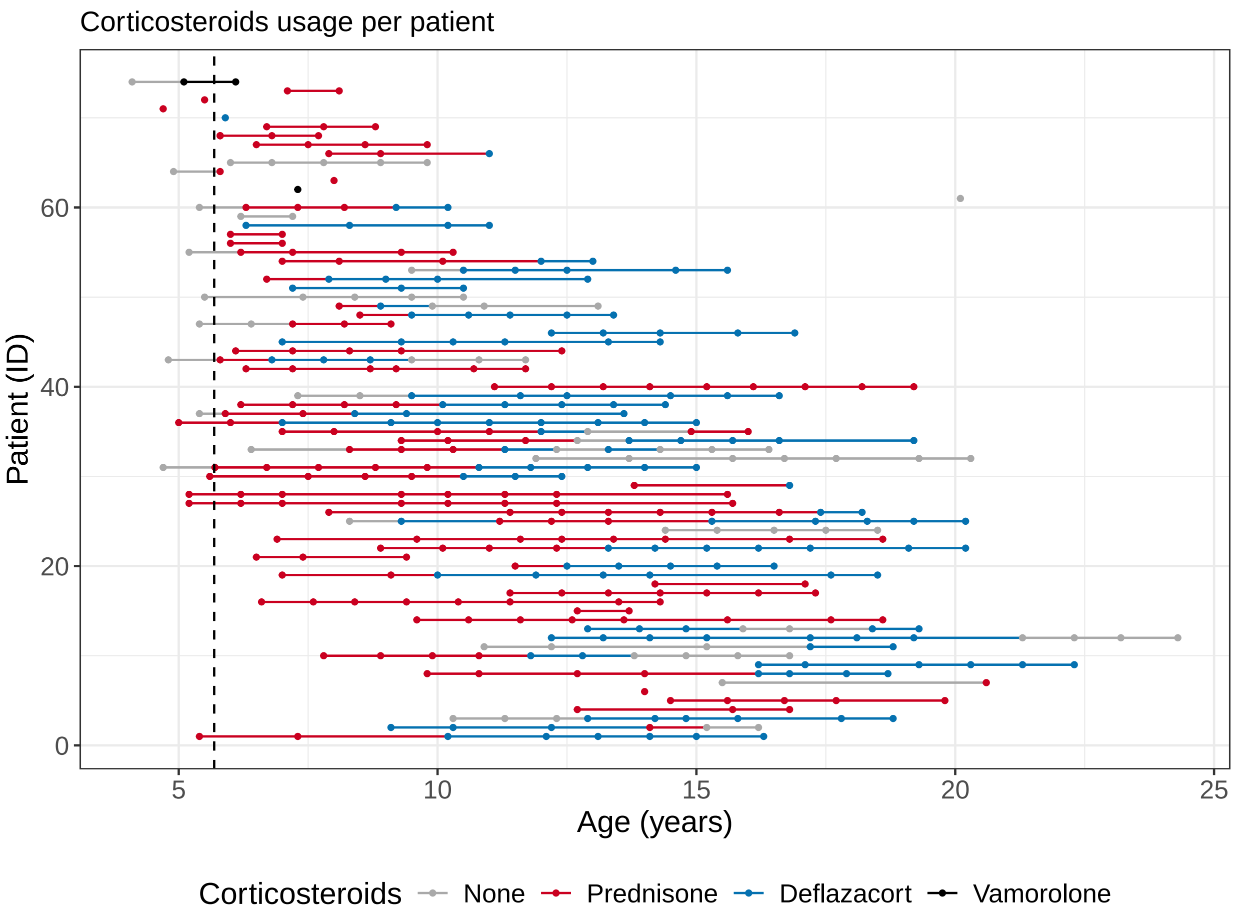

The biomarkers trajectories of the individual patients over treatment groups are:

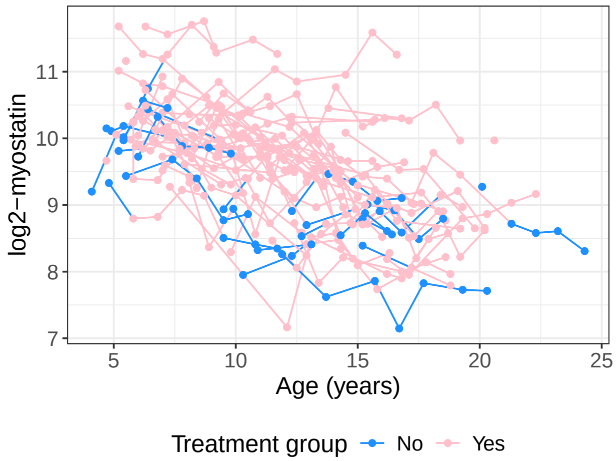

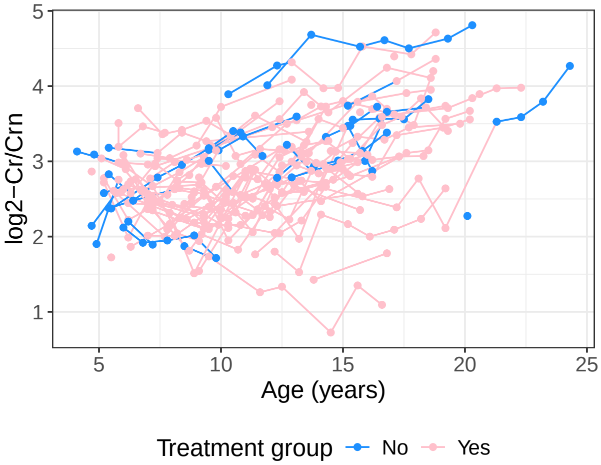

S3.1 Longitudinal progression

A spaghetti plot is presented for each functional score, where each line represents a patient and each point corresponds to a visit. This visualization allows us to observe both the overall trends in functional scores

over time and individual patient trajectories. For each functional score, a brief description and a summary table are provided, outlining the distribution of values. Additionally, a separate table reports the percentage of zero and missing values for each score, along with the number of patients who have at least one recorded observation for that specific test.

S3.1.1 Six-minute walk test

The 6-minute walk test (m) measures the distance an individual can walk in six minutes. The values of this functional score are strictly dependent of the time of loss ambulation: they are equal to zero on the first visit the patient is no longer able to move, and equal to NA afterword.

```
   Min. 1st Qu.  Median    Mean 3rd Qu.    Max.    NA's
      0      50     327     270     400     611     202
```

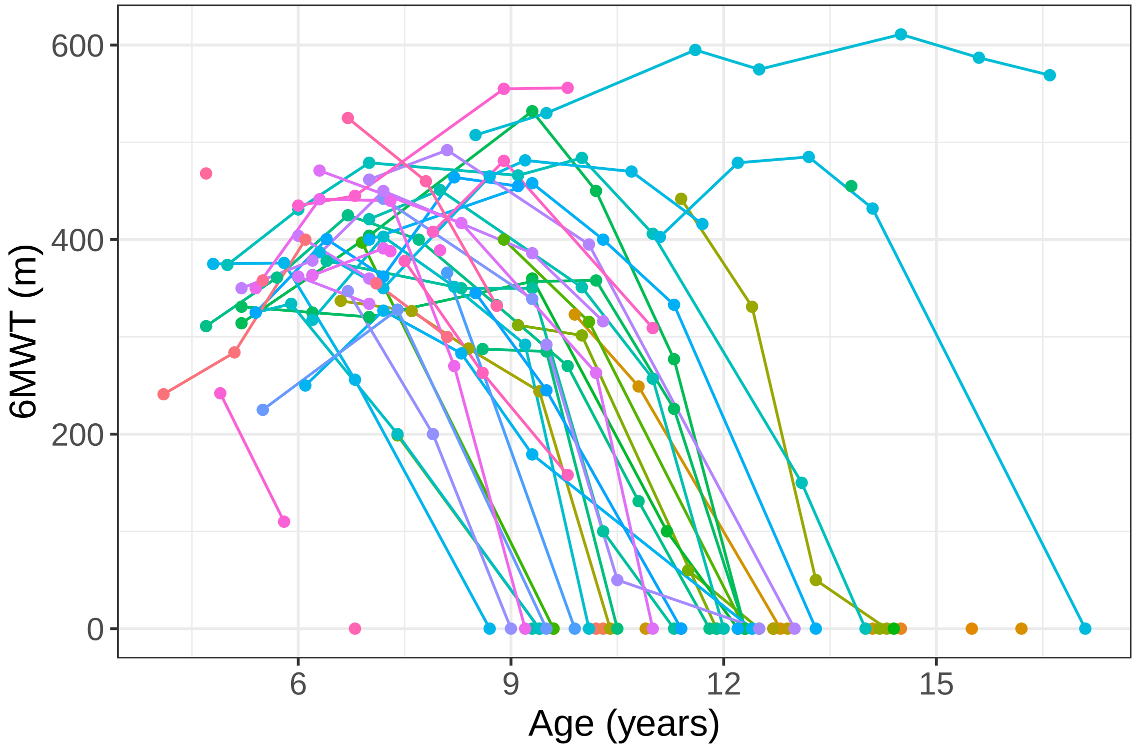

Metric	Value
Missing values (%)	50%
Zeros (%)	25%
Patients (n)	70

S3.1.2 North Star Ambulatory Assessment

The North Star Ambulatory Assessment (points) is a scale used to measure functional motor abilities in patients with neuromuscular disorders. It is a 17-item scale that assesses tasks such as standing, walking, and climbing stairs. Each task is scored from 0 (unable to perform) to 2 (normal performance), resulting in a total score ranging from 0 to 34.

```
Min. 1st Qu.  Median    Mean 3rd Qu.    Max.    NA's
 0.0     4.0    18.0    16.4    28.0    34.0     194
```

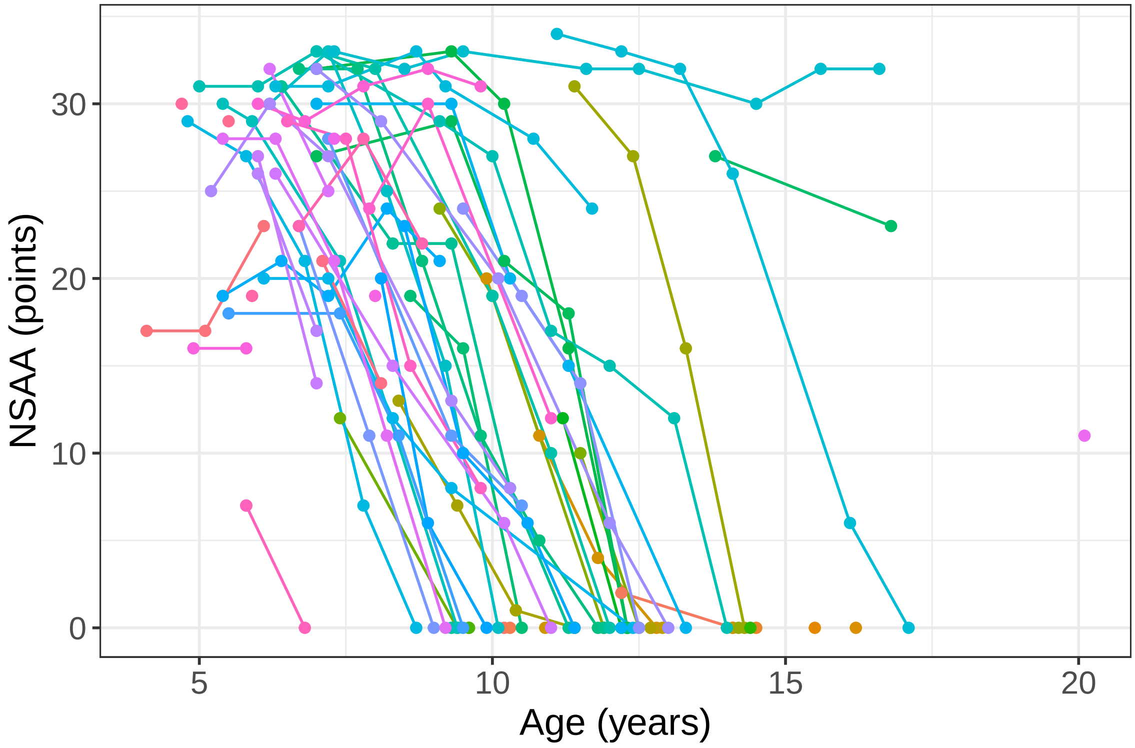

Metric	Value
Missing values (%)	48%
Zeros (%)	24%
Patients (n)	72

S3.1.3 10-minute walk/run test

The 10-meter run/walk test (m/s) measures the speed at which an individual can walk or run 10 meters. The values are equal to zero on the first visit the patient is no longer able to move, and equal to NA afterword.

```
Min. 1st Qu.  Median    Mean 3rd Qu.    Max.    NA's
0.00    0.74    1.56    1.36    2.12    3.03     180
```

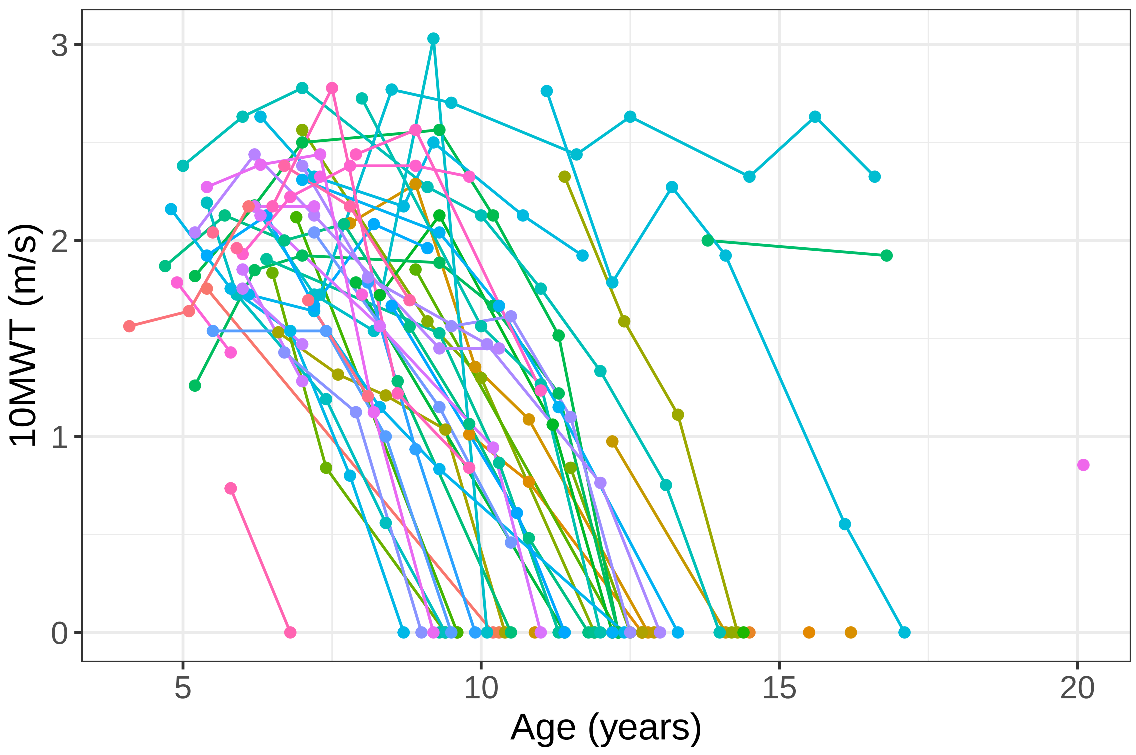

Metric	Value
Missing values (%)	45%
Zeros (%)	22%
Patients (n)	71

S3.1.4 PUL 2.0

The second version of the total score of the performance of the upper limb test (points). It includes 22 items that examine a range of movements, from lifting the arms to fine hand tasks, with a total score ranging from 0 to 42, where higher scores indicate better function. It is recorded in both ambulant and non-ambulant patients.

```
   Min. 1st Qu.  Median    Mean 3rd Qu.    Max.    NA's
    2.0    17.0    30.0    28.1    39.0    42.0     199
```

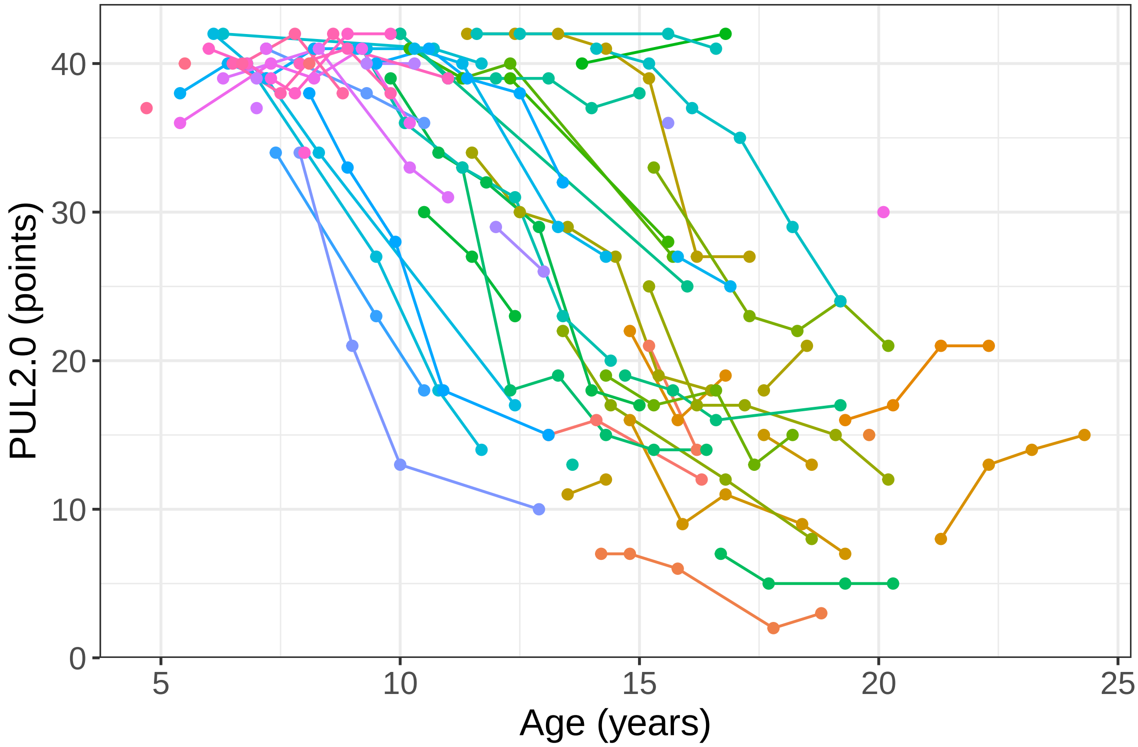

Metric	Value
Missing values (%)	49%
Zeros (%)	0%
Patients (n)	60

S3.2 Clinical milestones

At the start of the study, 14 patients had already lost ambulation and 9 had lost the ability to reach overhead. By the end, 23 patients remained ambulant and 32 retained overhead reach function. Additionally, 52 patients had not lost the hand-to-mouth function. Over the course of the study, 36 patients lost ambulation, 32 lost overhead reach function, and 21 lost hand-to-mouth function.

Table 12: Baseline

	No	Yes
Loss of ambulation	59	14
Loss of hand-to-mouth function	73	0
Loss of overhead-reach function	64	9

Table 13: End of Study

	No	Yes
Loss of ambulation	23	50
Loss of hand-to-mouth function	52	21
Loss of overhead-reach function	32	41

For each milestone, a survival plot is provided, illustrating the probability of not experiencing the event (e.g., loss of ambulation) over time. Accompanying each plot is a brief description and a summary table with quartiles, mean, median, minimum, and maximum ages at which the milestone was reached. The dashed red line in each plot indicates the median age at which the milestone occurred.

S3.2.1 Loss of ambulation

Inability to walk in a person who previously had the ability to walk.

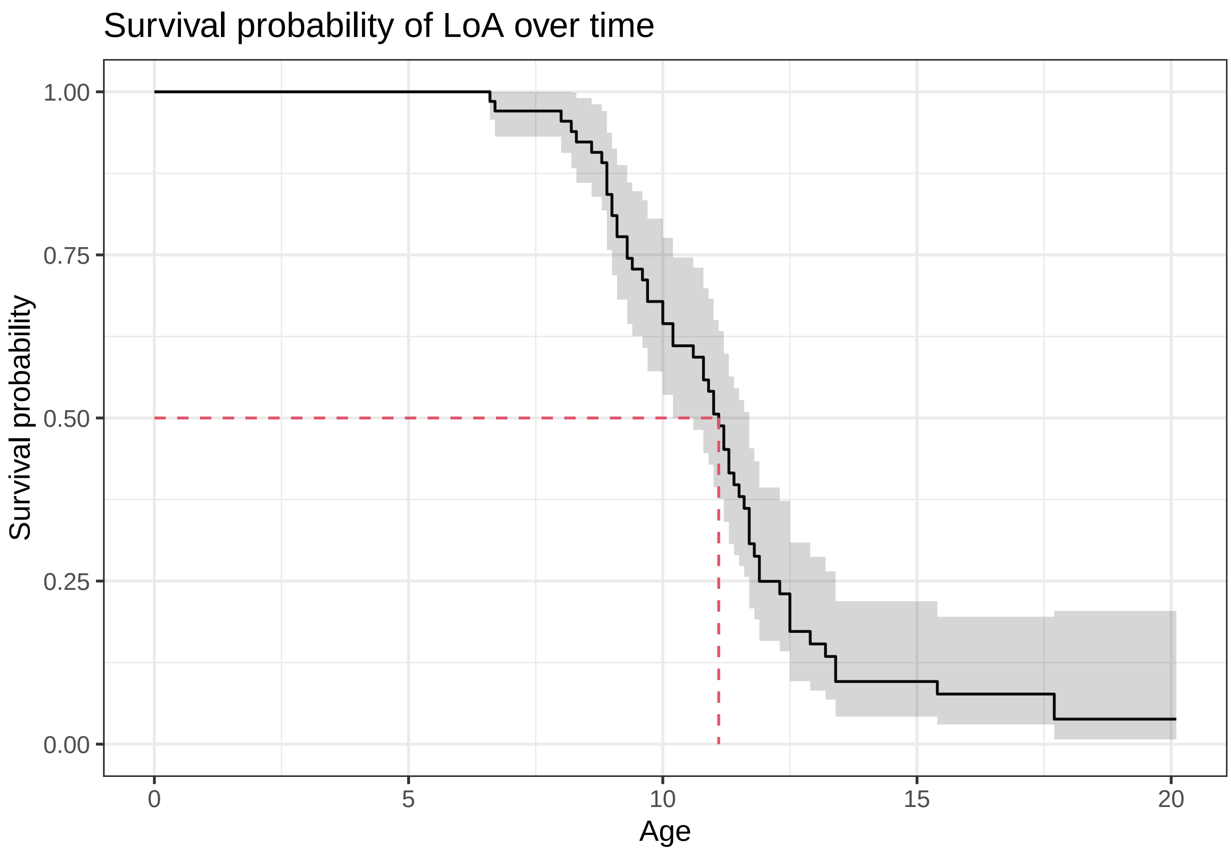


```
   Min. 1st Qu.  Median    Mean 3rd Qu.    Max.    NA's
    6.6     9.4    11.0    10.8    11.8    17.7      54
```

S3.2.2 Loss of overhead reach function

Inability to shoulder abduction and overhead reach. Hence, inability to reach things.

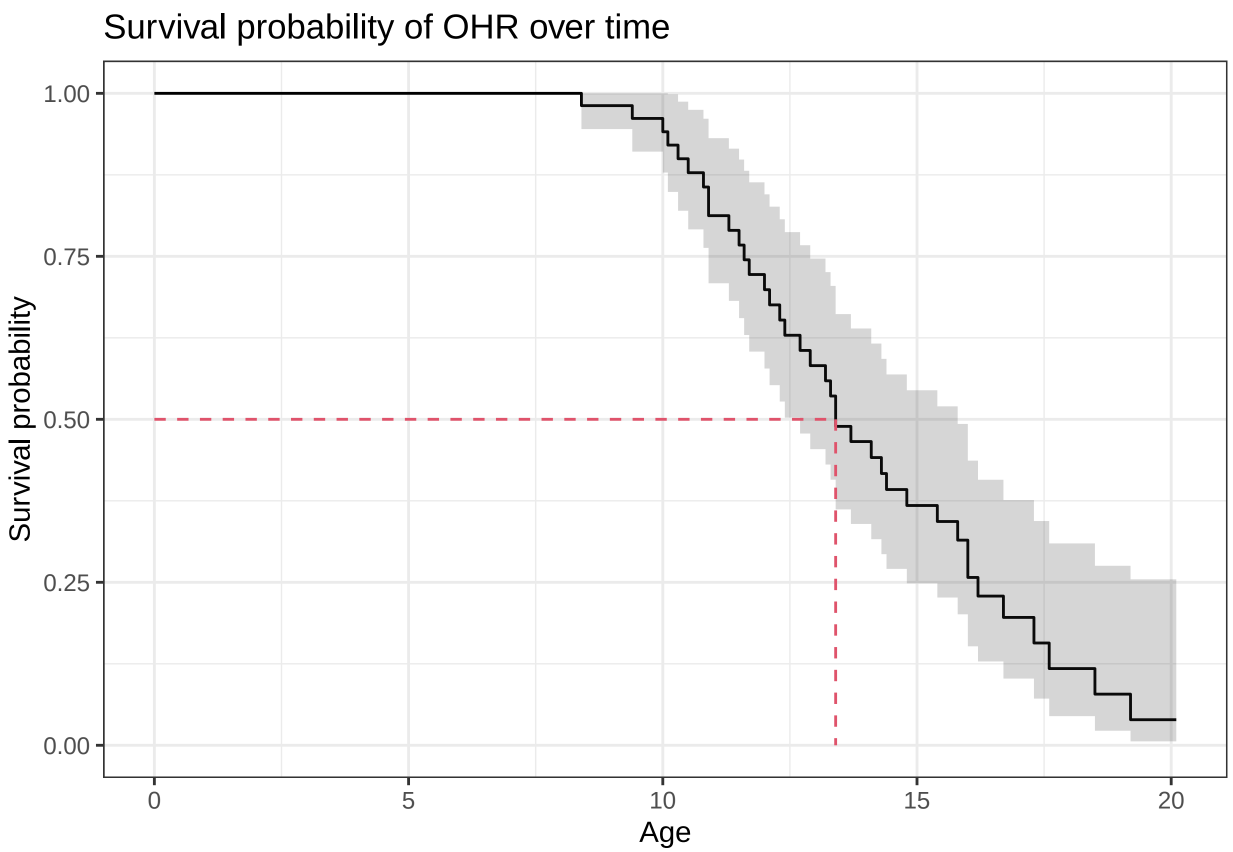


```
 Min. 1st Qu.  Median    Mean 3rd Qu.    Max.    NA's
  8.4    11.6    13.3    13.5    15.8    19.2     128
```

S3.2.3 Loss of hand to mouth function

Inability to move and to reach the mouth. Hence, inability to self-feed.

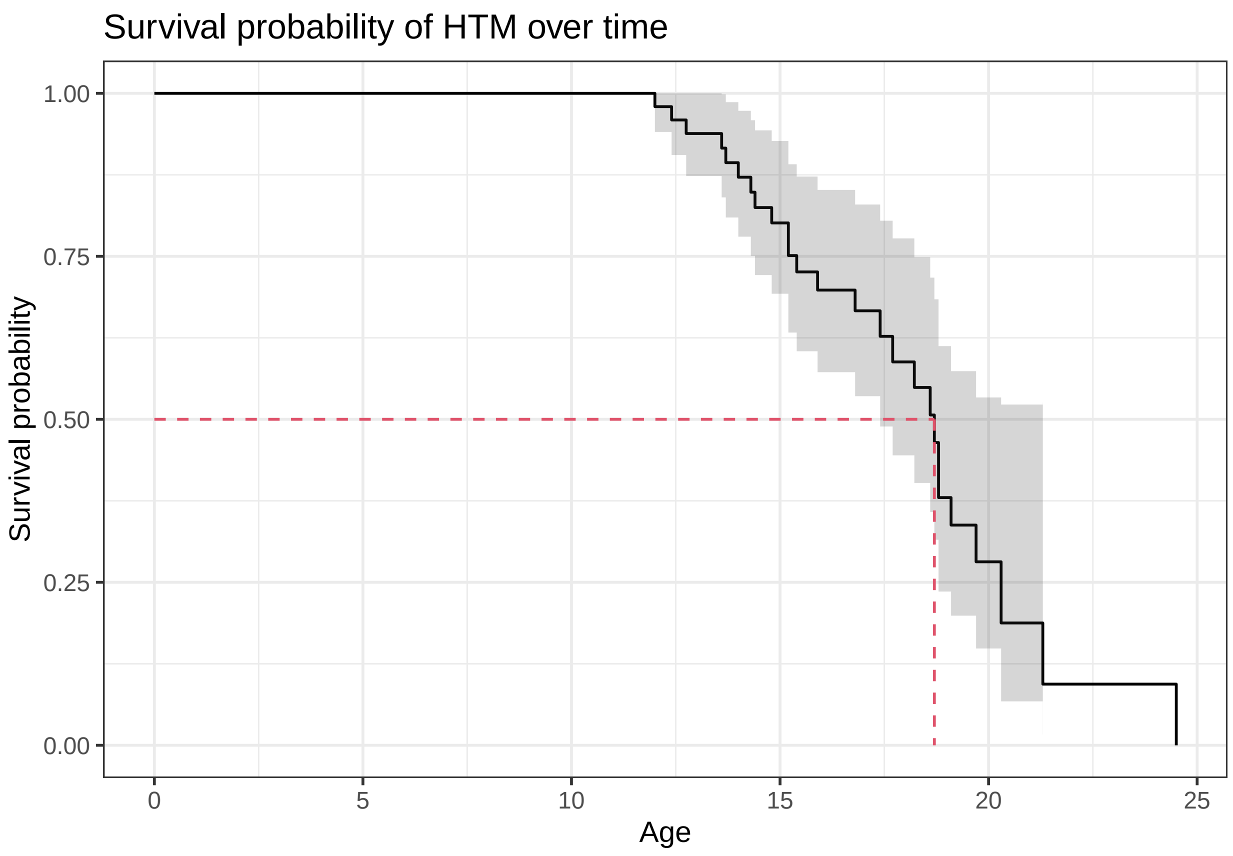

Min.	1st Qu.	Median	Mean	3rd Qu.	Max.	NA's
12.0	14.3	16.8	16.7	18.8	24.5	228

S4 Association of functional scores and clinical milestones with CS treatment

Table 14: Model with 10MWT

Predictors	Estimates	p.values
Intercept	0.294	0.063
Age (years)	-0.190	< 0.001
Treatment (yes)	0.387	0.006
BMI (kg/m^2)	-0.073	< 0.001

Table 15: Model with 6MWT

Predictors	Estimates	p.values
Intercept	65.56	0.053
Age (years)	-26.92	< 0.001
Treatment (yes)	87.96	0.004
BMI (kg/m^2)	-18.44	< 0.001

Table 16: Model with NSAA

Predictors	Estimates	p.values
Intercept	2.041	0.339
Age (years)	-2.885	< 0.001
Treatment (yes)	4.701	0.011
BMI (kg/m^2)	-0.973	< 0.001

Table 17: Model with PUL2.0

Predictors	Estimates	p.values
Intercept	24.387	< 0.001
Age (years)	-1.987	< 0.001
Treatment (yes)	6.891	< 0.001
BMI (kg/m^2)	-0.366	0.002

Table 18: Model of LoA

Predictors	Hazard Ratios	p-values
Treatment (yes)	0.253	0.002
BMI (kg/m^2)	1.035	0.258

Table 19: Model of OHR

Predictors	Hazard Ratios	p-values
Treatment (yes)	0.492	0.112
BMI (kg/m^2)	0.995	0.904

Table 20: Model of HTM

Predictors	Hazard Ratios	p-values
Treatment (yes)	0.378	0.162
BMI (kg/m^2)	0.965	0.497

S5 Association of biomarkers with age and CS treatment

Table 21: Model with log2-myostatin

Predictors	Estimates	p.values
Intercept	9.204	< 0.001
Age (years)	-0.098	< 0.001
Treatment (yes)	0.462	< 0.001

Table 22: Model with log2-Cr/Crn

Predictors	Estimates	p.values
Intercept	3.192	< 0.001
Age (years)	0.084	< 0.001
Treatment (yes)	-0.343	< 0.001

S6 Association of the functional tests with the biomarkers

Here we show the results for 6MWT and 10MWT. The results of NSAA and PUL2.0 are in the manuscript.

Table 23: Comparison of the models between 10MWT with only myostatin, with only ratio and with both of them

	Only myostatin		Only ratio		Both	
Predictors	Estimates	p-values	Estimates	p-values	Estimates	p-values
Intercept	0.563	0.001	0.479	< 0.001	0.530	< 0.001
Age (years)	-0.157	< 0.001	-0.152	< 0.001	-0.142	< 0.001
Treatment (yes)	0.091	0.56	0.073	0.561	0.046	0.731
BMI (kg/m^2)	-0.067	< 0.001	-0.063	< 0.001	-0.058	< 0.001
log2-myostatin	0.353	< 0.001			0.096	0.231
log2-Cr/Crn			-0.893	< 0.001	-0.828	< 0.001

Table 24: Comparison of the models between 6MWT with only myostatin, with only ratio and with both of them

	Only myostatin		Only ratio		Both	
Predictors	Estimates	p-values	Estimates	p-values	Estimates	p-values
Intercept	127.25	< 0.001	83.46	0.004	95.42	0.002
Age (years)	-17.41	0.001	-21.30	< 0.001	-19.37	< 0.001
Treatment (yes)	28.04	0.408	44.80	0.09	36.18	0.201
BMI (kg/m^2)	-16.49	< 0.001	-15.96	< 0.001	-15.33	< 0.001
log2-myostatin	83.58	< 0.001			13.76	0.424
log2-Cr/Crn			-190.12	< 0.001	-180.99	< 0.001

S7 Association of the clinical milestones with the biomarkers

The table of estimates of the model with loss of ambulation is in the manuscript. Here are shown the same results for the hand-to-mouth function and the overhead reach function.

Table 25: Comparison of the models between OHR with only myostatin, with only ratio and with both of them

	Only myostatin		Only ratio		Both	
Predictors	Hazard Ratios	p-values	Hazard Ratios	p-values	Hazard Ratios	p-values
Treatment (yes)	0.805	0.631	1.653	0.309	1.641	0.321
BMI (kg/m^2)	0.986	0.729	1.024	0.566	1.024	0.576
log2-myostatin	0.445	< 0.001			0.969	0.926
log2-Cr/Crn			7.171	< 0.001	6.895	0.002

Table 26: Comparison of the models between HTM with only myostatin, with only ratio and with both of them

	Only myostatin		Only ratio		Both	
Predictors	Hazard Ratios	p-values	Hazard Ratios	p-values	Hazard Ratios	p-values
Treatment (yes)	0.561	0.425	0.659	0.583	0.678	0.614
BMI (kg/m^2)	1.004	0.937	1.019	0.721	1.021	0.700
log2-myostatin	0.367	0.057			0.819	0.796
log2-Cr/Crn			4.188	0.018	3.553	0.149

S8 Association with creatinine

Because creatine is not always easy to obtain in the lab, we also briefly checked the association between the functional scores and clinical milestones with only creatinine. It has already been proven in previous studies that the serum levels of creatinine decrease in neuromuscular diseases such as DMD, BMD (Wang et al. 2021), and spinal muscular atrophy (Alves et al. 2020). Moreover, it has been demonstrated that creatinine reflects disease severity in dystrophinopathy, and may be used to distinguish DMD from BMD patients (Zhang et al. 2015).

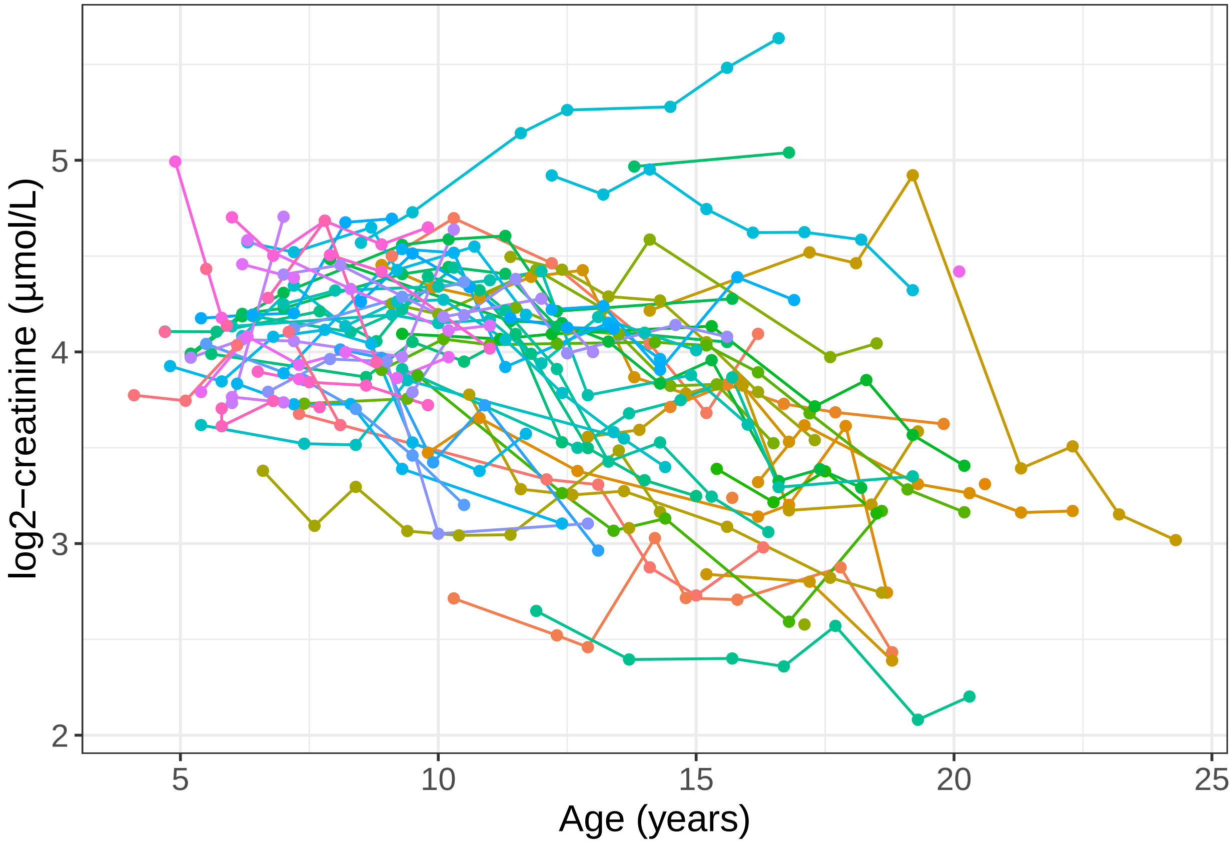

Table 27: Model between 10MWT and log2-creatinine

Predictors	Estimates	p.values
Intercept	-3.384	< 0.001
Age (years)	-0.180	< 0.001
Treatment (yes)	0.041	0.761
BMI (kg/m^2)	-0.065	< 0.001
log2-creatinine	0.989	< 0.001

Table 28: Model between 6MWT and log2-creatinine

Predictors	Estimates	p.values
Intercept	-712.71	< 0.001
Age (years)	-24.82	< 0.001
Treatment (yes)	34.99	0.221
BMI (kg/m^2)	-16.41	< 0.001
log2-creatinine	206.37	< 0.001

Table 29: Model between NSAA and log2-creatinine

Predictors	Estimates	p.values
Intercept	-40.167	< 0.001
Age (years)	-2.662	< 0.001
Treatment (yes)	0.550	0.77
BMI (kg/m^2)	-0.847	< 0.001
log2-creatinine	11.515	< 0.001

Table 30: Model between PUL2.0 and log2-creatinine

Predictors	Estimates	p.values
Intercept	-9.955	0.006
Age (years)	-1.336	< 0.001
Treatment (yes)	4.312	< 0.001
BMI (kg/m^2)	-0.221	0.022
log2-creatinine	9.207	< 0.001

Table 31: Model between LoA and log2-creatinine

Predictors	Hazard Ratios	p-values
Treatment (yes)	0.596	0.271
BMI (kg/m^2)	1.057	0.086
log2-creatinine	0.219	< 0.001

Table 32: Model between OHR and log2-creatinine

Predictors	Hazard Ratios	p-values
Treatment (yes)	2.049	0.156
BMI (kg/m^2)	1.031	0.448
log2-creatinine	0.097	< 0.001

Table 33: Model between HTM and log2-creatinine

Predictors	Hazard Ratios	p-values
Treatment (yes)	0.678	0.606
BMI (kg/m^2)	1.035	0.550
log2-creatinine	0.241	0.031

References

Alves, Christiano R. R., Ren Zhang, Alec J. Johnstone, Reid Garner, Pann H. Nwe, Jennifer J. Siranosian, and Kathryn J. Swoboda. 2020. "Serum Creatinine Is a Biomarker of Progressive Denervation in Spinal Muscular Atrophy." *Neurology* 94 (9). https://doi.org/10.1212/wnl.0000000000008762.

Cohen, Jacob. 1988. *Statistical Power Analysis for the Behavioral Sciences*. 2nd ed. Hoboken: Taylor; Francis.

McDonald, Craig M., Erik K. Henricson, R. Ted Abresch, Julaine Florence, Michelle Eagle, Eduard Gappmaier, Allan M. Glanzman, et al. 2013. "The 6-Minute Walk Test and Other Clinical Endpoints in Duchenne Muscular Dystrophy: Reliability, Concurrent Validity, and Minimal Clinically Important Differences from a Multicenter Study." *Muscle & Nerve* 48 (3): 357–68. https://doi.org/10.1002/mus.23905.

Wang, Liang, Min Xu, Dawei Liu, Yingyin Liang, Pinning Feng, Huan Li, Yuling Zhu, et al. 2021. "Serum Creatinine as a Biomarker for Dystrophinopathy: A Cross-Sectional and Longitudinal Study." *BMC Neurology* 21 (1). https://doi.org/10.1186/s12883-021-02382-7.

Zhang, Huili, Yuling Zhu, Yiming Sun, Yingyin Liang, Yaqin Li, Yu Zhang, Langhui Deng, Xingxuan Wen, and Cheng Zhang. 2015. "Serum Creatinine Level: A Supplemental Index to Distinguish Duchenne Muscular Dystrophy from Becker Muscular Dystrophy." *Disease Markers* 2015: 1–5. https://doi.org/10.1155/2015/141856.
